# Whole genome sequencing provides evidence for *Bacillus velezensis* SH-1471 as a beneficial rhizosphere bacterium in plants

**DOI:** 10.1038/s41598-023-48171-9

**Published:** 2023-11-27

**Authors:** Yunxin Shen, Zhufeng Shi, Jiangyuan Zhao, Minggang Li, Jiacai Tang, Nan Wang, Yanfang Mo, Tongyu Yang, Xudong Zhou, Qibin Chen, Peiweng Yang

**Affiliations:** 1 Institute of Agricultural Environment and Resources, Yunnan Academy of Agricultural Sciences, Kunming, 650204 China; 2 College of Plant Protection, Yunnan Agricultural University, Kunming, 655508 China; 3 Yunnan Institute of Microbiology, Yunnan University, Kunming, 650106 China

**Keywords:** Microbiology, Bacteria, Bacterial genomics

## Abstract

*Bacillus* is widely used in agriculture due to its diverse biological activities. We isolated a *Bacillus velezensis* SH-1471 from the rhizosphere soil of healthy tobacco, which has broad-spectrum antagonistic activity against a variety of plant pathogenic fungi such as *Fusarium oxysporum*, and can be colonized in the rhizosphere of a variety of plants. This study will further explore its mechanism by combining biological and molecular biology methods. SH-1471 contains a ring chromosome of 4,181,346 bp with a mean G + C content of 46.18%. We identified 14 homologous genes related to biosynthesis of resistant secondary metabolite, and three clusters encoded potential new antibacterial substances. It also contains a large number of genes from colonizing bacteria and genes related to plant bacterial interactions. It also contains genes related to environmental stress, as well as genes related to drug resistance. We also found that there are many metabolites in the strain that can inhibit the growth of pathogens. In addition, our indoor pot test found that SH-1471 has a good control effect on tomato wilt, and could significantly improve plant height, stem circumference, root length, root weight, and fresh weight and dry weight of the aboveground part of tomato seedlings. Therefore, SH-1471 is a potential biological control strain with important application value. The results of this study will help to further study the mechanism of SH-1471 in biological control of plant diseases and promote its application.

## Introduction

Diseases caused by soil borne pathogens usually lead to root rot, growth retardation, and seedling wilt in plants that have been invaded by roots^1^. As a result, the yield and quality of important cash crop have declined significantly, which has highlighted international food safety and environmental security issues^2^. Therefore, agricultural departments in various countries are also paying more and more attention to the use of biological fertilizers and biological control agents. In this process, biological control agents, as substitutes for chemical pesticides, have a very broad application prospect in sustainable agriculture.

*Bacillus* is a ubiquitous bacterium widely distributed in the natural environment, especially in the rhizosphere and plant roots^3^. This genus of strains produces various secondary metabolites with antibacterial activity and secretes various compounds that promote plant growth^4^. And they can produce heat resistant and dry resistant endospore, which are easy to store and transport as stable products. In previous studies, bacillus strains produced a variety of secondary metabolites beneficial to plants, including lipopeptides synthesized by non-ribosome peptide synthesis (NRPS), polyketide syntheses (PKS)^5^, and linear azol (In) E-containing peptides (LAP), bacteriocin, thiopeptide, terpene, etc. synthesized by ribosome peptide synthesis (RPS)^6^. At the same time, it also has secondary metabolites that trigger induced systemic resistance (ISR), exhibiting biological activity against various plant pathogens, thereby protecting plants from pathogen attacks^7^. In addition to these activities, bacillus has also been reported to use growth promoting substances such as auxin, cytokinin and gibberellin to promote plant growth^8^. For example, *B. velezensis* FZB42 can produce Indole-3-acetic acid (IAA) and cytokinin, both of which are related to promoting plant growth^9^.

The colonization of Bacillus in plant roots is the key to biological control and the main factor of stable performance in the field^10^. Root colonization is divided into two steps: chemotaxis towards the root and subsequent formation of biofilms on the root surface, and chemotaxis induction enhances the colonization and beneficial effects of *B. subtilis* strains that promote plant growth in response to root exudates^11^. According to previous studies, colonization and biofilm formation are closely related to the biological control of bacillus, and many genes related to biofilm formation and colonization have been proved to play an important role in biological control, including *motA*, *motB* and *flgM* genes related to flagella movement^12^, and the genes of Bacillus specific biofilm formation pathway *kinB*, *spo0A*, *spo0F*, *degU* and *degS*^13^.

Whole genome sequencing is an effective method to more comprehensively understand the technical characteristics and safety of strains at the gene level, which helps to understand the biological control mechanism of biocontrol strains, and provides valuable information for the application of these microorganisms. There are various types of beneficial secondary metabolites secreted by bacillus, and bacillus isolated from different environments have high genetic diversity^14^. Therefore, systematic exploration of the genetic basis of biological control activity of biocontrol strains is an important basis for understanding and using biocontrol strains. This study isolated culturable microorganism from the rhizosphere of healthy tobacco plants with high incidence of tobacco bacterial wilt in Yunnan Province. Their antagonistic and diverse biological activities against pathogenic microorganisms were measured in laboratory, and their taxonomic status and biological activities were determined by whole genome sequencing technology and genome mining method. Non-targeted metabolomics technology was used to detect the types and contents of metabolites related to antibacterial activity in the fermentation broth of strains. The purpose of this study was to provide efficient microbial strain resources for the biological control of plant diseases and the promotion of plant growth, and to provide theoretical basis for its popularization and application.

## Results

### Inhibition effect of strain SH-1471 on plant pathogenic fungi

**Figure 1:**
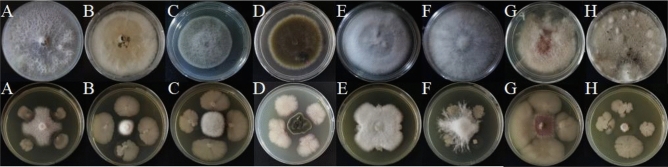
Antagonistic effect of *Bacillus velezensis* SH-1471 on plant pathogenic fungi. **(A)** *Fusarium oxysporum*. **(B)** *Phoma matteuciicola*. **(C)** *Exserohilum turcicum*. **(D)** *Alternaria alternate*. **(E)** *Colletotrichum micotianae*. **(F)** *Diaporthe eres*. **(G)** *Phytophthora parasitica*. **(H)** *Sclerotinia sclerotiorum*. The upper panel represents the pathogenic fungus con-trol experiment, the lower panel represents the antagonistic antibacte-rial and antibacterial experimental bacteria.

**Figure 2:**
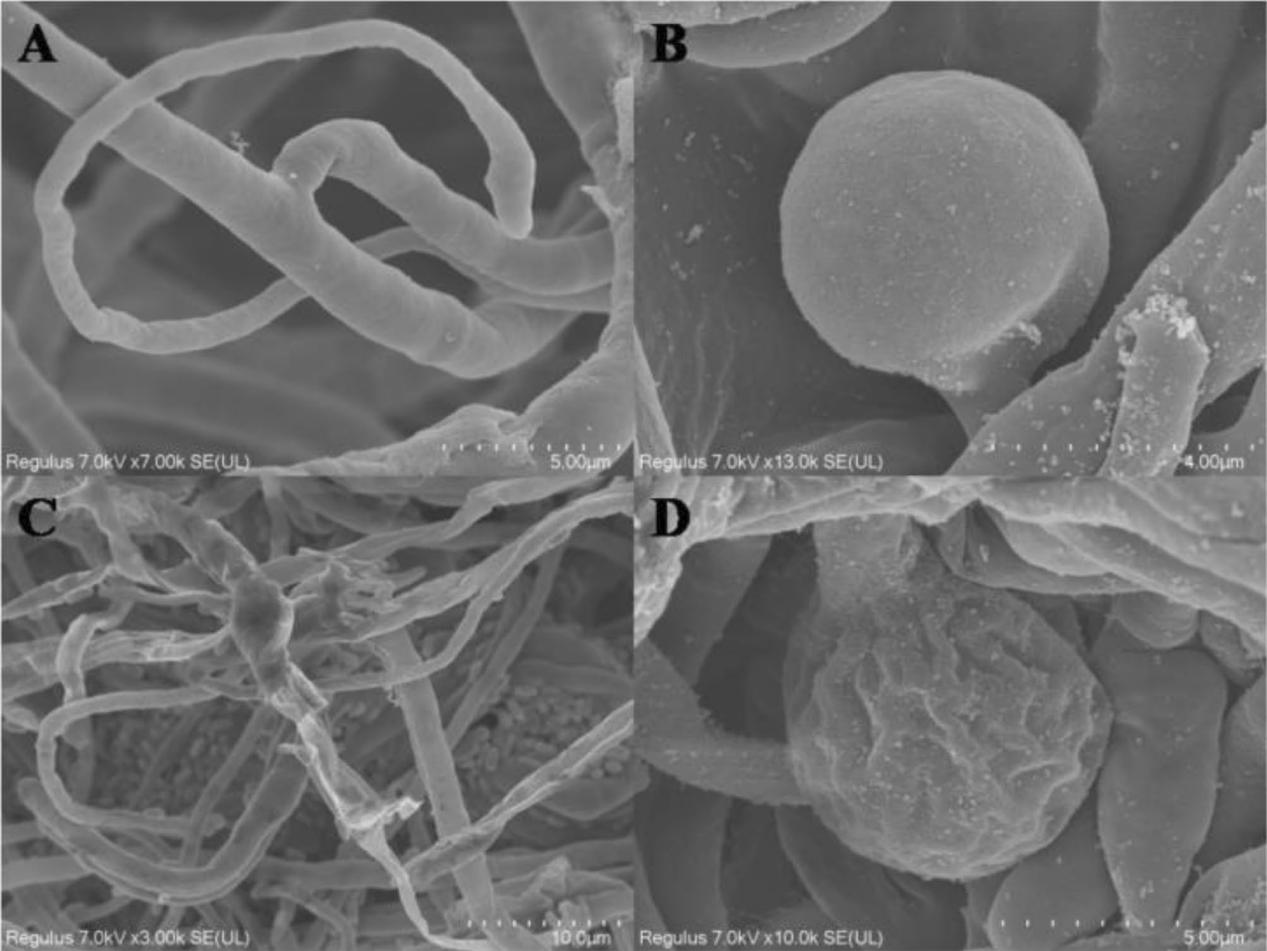
Effect of strain SH-1471 on the growth of *Fusarium oxysporum.* (**A,B**) SEM image of *F. oxysporum* mycelium in the control group. (**C,D**) SEM image of *F. oxysporum* mycelium in treatment group.

The results of the plate confrontation experiment showed that *B. velezensis* SH-1471 had good inhibitory effects on various pathogenic microorganisms (Fig. 1). Specifically, our experiment showed that its inhibition rates on *Sclerotinia scrotiorum*, *Phoma mateuciicola*, and *Fusarium oxysporum* were 93.5%, 90.3%, and 88.6%(Table 1). In addition, we observed by scanning electron microscopy that the mycelia of *F. oxysporum* in the inhibition area would appear passivation, bending and thinning(Fig. 2). The results indicated that SH-1471 could inhibit fungal growth by distorting the new mycelium during mycelial growth.

**Table 1:** Antagonistic effect of *Bacillus velezensis* SH-1471 against eight phytopathogens.

Pathogenic fungi	Diameter of zone of inhibition (mm)
*Fusarium oxysporum*	88.6 ± 0.65b
*Phoma matteuciicola*	90.3 ± 0.85b
*Exserohilum turcicum*	81.5 ± 0.76d
*Alternaria alternate*	83.2 ± 0.99d
*Colletotrichum micotianae*	65.9 ± 0.66e
*Diaporthe eres*	86.9 ± 0.29c
*Phytophthora parasitica*	88.5 ± 0.58b
*Sclerotinia sclerotiorum*	93.5 ± 0.49a

### Determination of biofilm formation and plant growth-promoting traits of SH-1471

**Figure 3:**
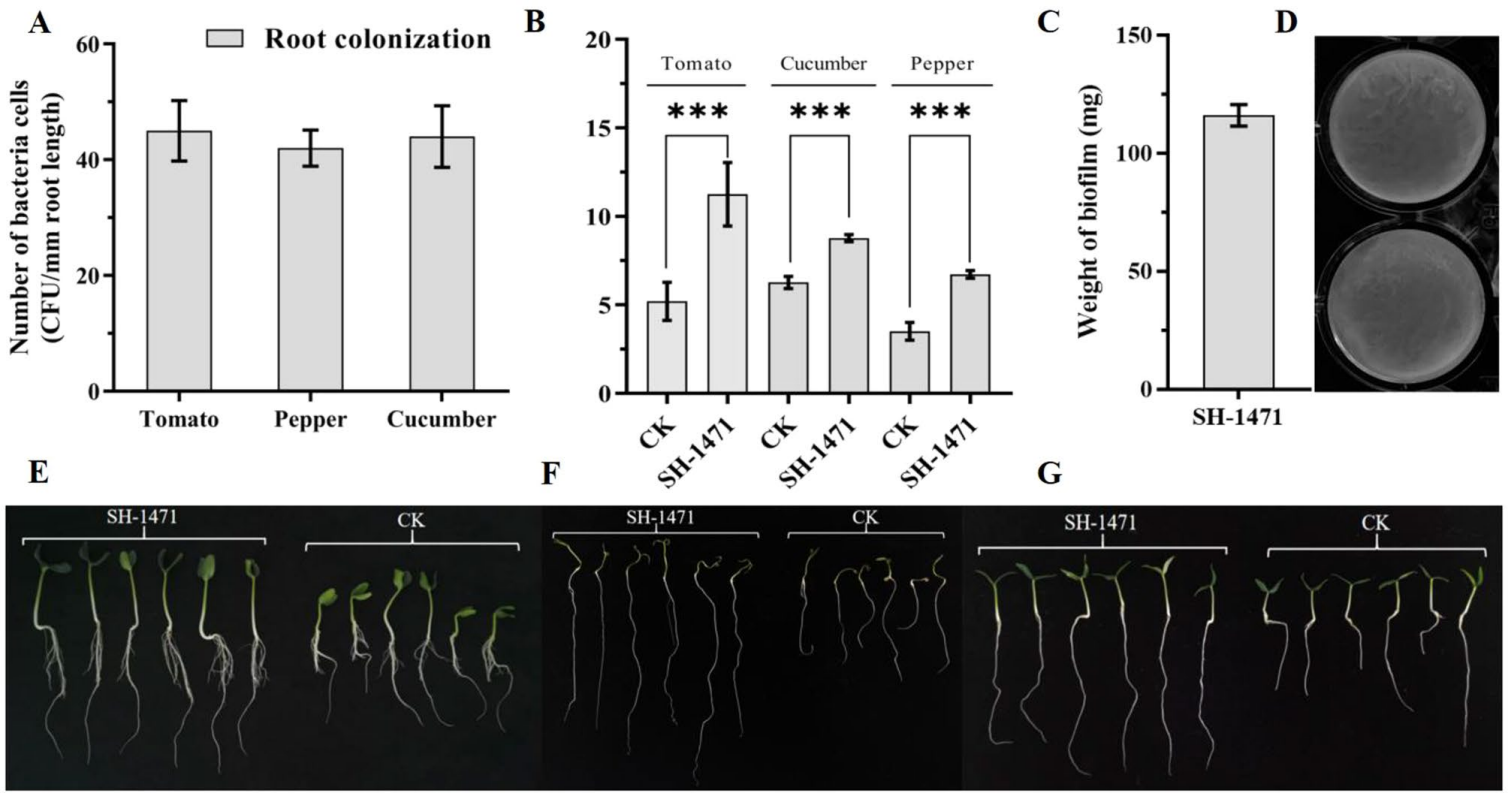
Determination of biofilm formation, colonization and root promoting ability of strain SH-1471 in plant roots. **(A)** The colonization ability of strain SH-1471 in plant roots. **(B)** The promoting effect of strain SH-1471 on the root growth of tomato seedlings. **(C)** Weight of biofilm formed by *Bacillus velezensis* SH-1471 after 16 h of static cultivation. **(D)** The biofilm formed by *Bacillus velezensis* SH-1471 on a 48 well plate. **(E)** The root length of cucumber seedlings. **(F)** The root length of tomato seedlings. **(G)** The root length of pepper seedlings. Triple asterisk indicates significant differences between treatments (*p* < 0.001).

The formation of biofilms is an important factor determining the functional strains in plant roots. We conducted qualitative and quantitative analysis on the biofilm formation ability of *B. velezensis* SH-1471 using MSgg culture medium. 16 h after inoculation, *B. velezensis* SH-1471 can form a large number of folds on MSgg medium(Fig. 3D). After measurement, *B. velezensis* SH-1471 can form a biofilm of 116 mg(Fig. 3C). In this study, we tested the colonization ability of *B. velezensis* SH-1471 on tomato, cucumber, and chili roots, and the results showed that *B. velezensis* SH-1471 can colonize the root surface of many plants(Fig. 3A). In addition, through experiments promoting plant growth, we found that *B.velezensis* SH-1471 can significantly promote the development of tomato roots(Fig. 3B, E, F, G). The above results indicate that *B. velezensis* SH-1471 can not only effectively colonize the rhizosphere of plants, but also promote root growth, greatly improving the efficiency of the beneficial effects of *B. velezensis* SH-1471 on plants.

### Results of functional assays of strain SH-1471

The functional screening results showed that strain SH-1471 also had the ability to produce proteases, cellulase, dissolve inorganic phosphorus, nitrogen fixation, siderophore, and secretion of amylase(Supplementary Fig S1). In summary, this strain not only has great application prospects in crop disease biological control, but also has broad application prospects in promoting crop growth and providing soil fertility.

### Identification of strain SH-1471

**Figure 4:**
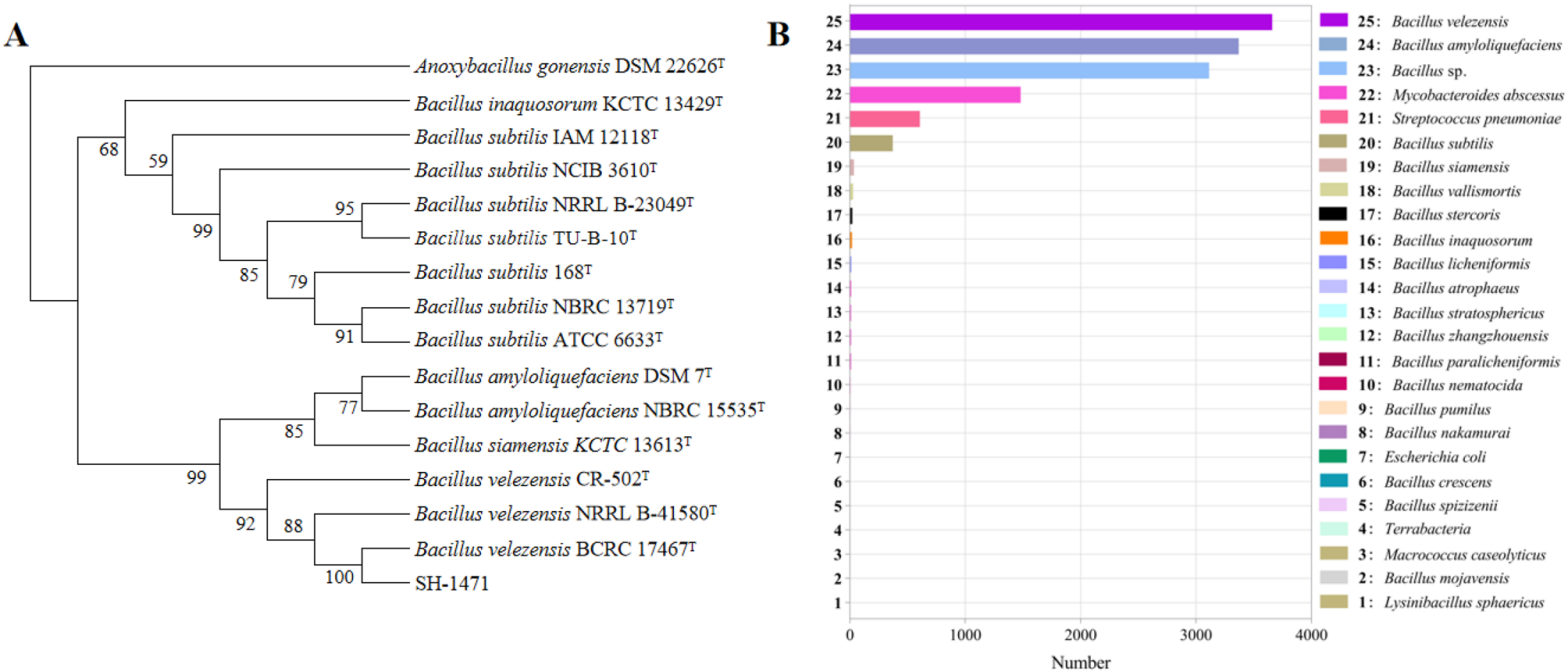
Identification of *Bacillus velezensis* SH-1471 strain. **(A)** Phylogenetic tree based on nucleotide sequence of 16S rRNA, *gyrA* and *gyrB* gene. **(B)** NCBI nr database.

The strains SH-1471 spores are rod-shaped, the center of the colony is milky white, no pigment production, the colony shape is irregular, the edges are radial, the surface of the colony is viscous, slightly convex (Supplementary Fig S2). Numerous studies have shown that the differentiation between *B. subtilis*, *B. amyloliquefaciens*, and *B. velezensis* cannot solely rely on the 16S rRNA gene sequence. We conducted phylogenetic analysis of *B. velezensis* SH-1471 using the maximum likelihood method based on three butler genes (16S rRNA, *gyrA*, and *gyrB*). The results showed that the strain SH-1471 had the highest homology with *B. velezensis* BCRC 17467, followed by B-41580 and CR-502 (Fig. 4A). We also used dDDH and ANI to analyze the SH-1471 genome for reference genome differences from the genus *Bacillus* (Supplementary Table 1). In addition, the gene sequence of strain SH-1471 was compared with the NCBI nr database, and 4172 genes in the genome were annotated on the NCBI nr database. The top 25 genes are shown in Fig. 3B, and the amplified sequence of strain SH-1471 is the most similar to that of *B. velezensis* (Fig. 4B).

### Genome sequencing and analysis of strain SH-1471

**Figure 5:**
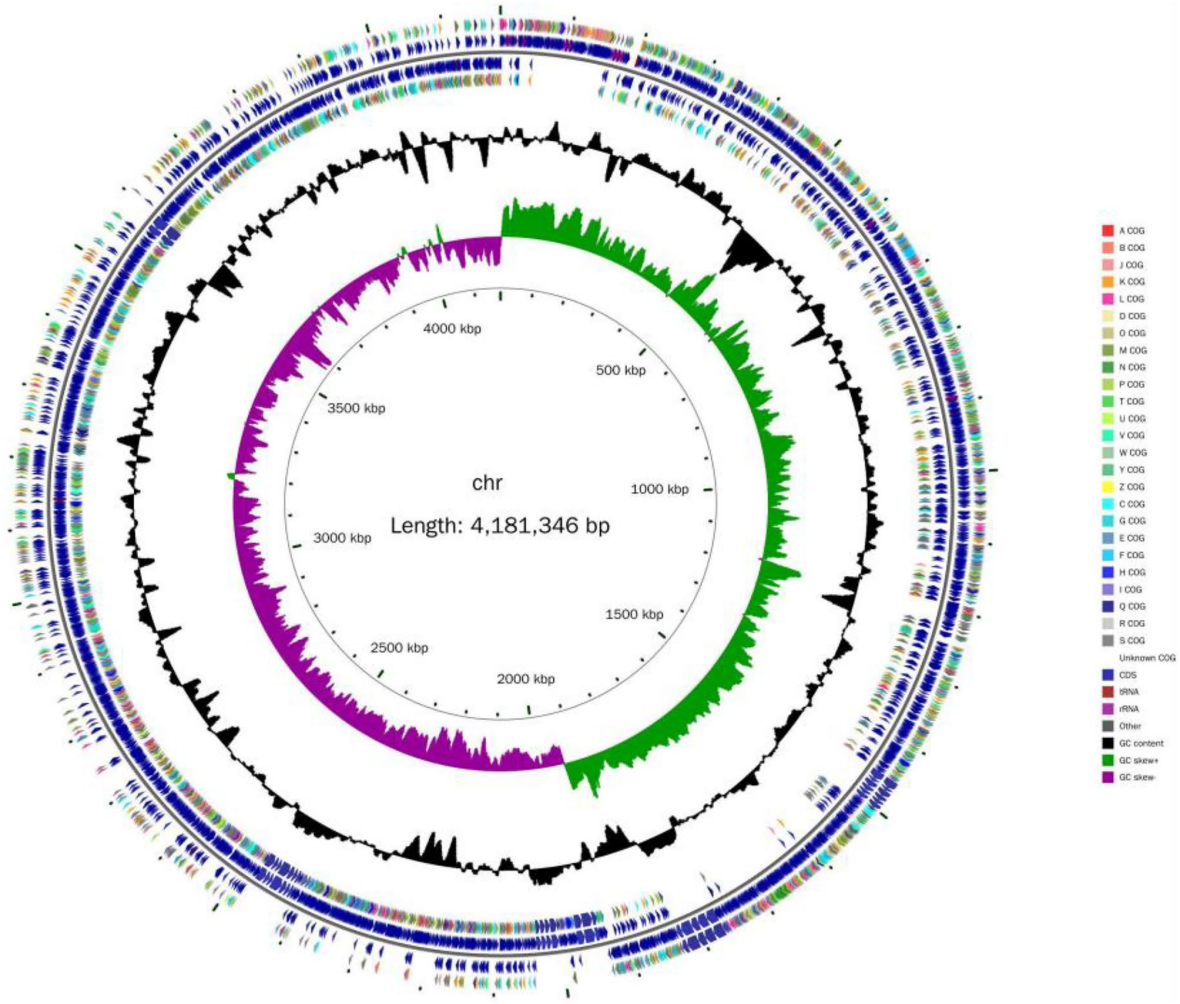
Genome circle map of *Bacillus velezensis* SH-1471. From the inside to the outside, the first circle represents the scale; The second lap represents GC Skew; The third circle represents the GC content; The fourth and seventh circles represent the COG to which each CDS belongs; The fifth and sixth circles represent the position of CDS, tRNA, and RNA on the genome.

**Figure 6:**
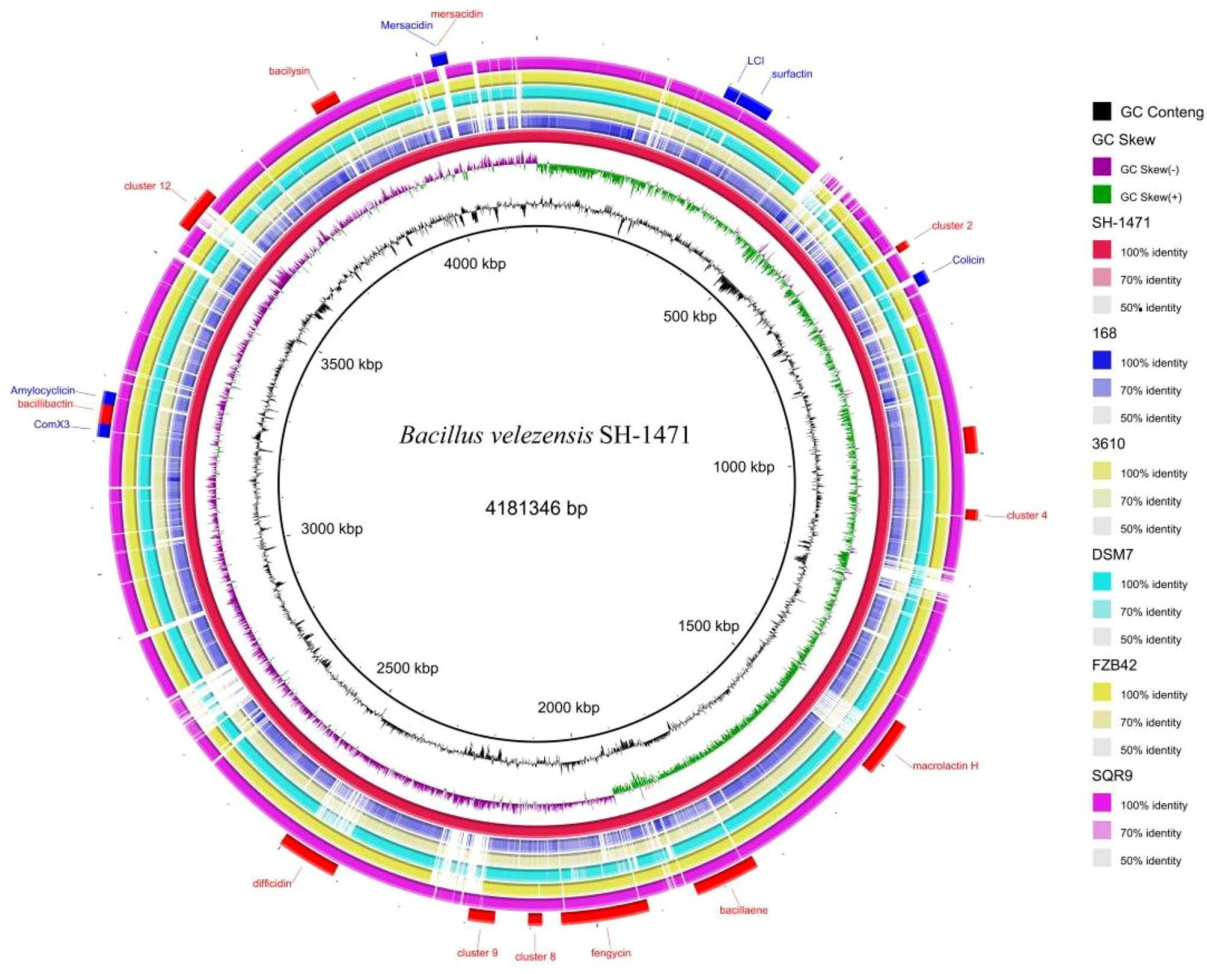
*Bacillus velezensis* SH-1471 genomic characteristics and comparative genomic circles. The graph shows from inside out: **(1)** GC Content, **(2)** GC Skew, **(3)** *Bacillus velezensis* SH-1471 genome, **(4–8)** comparison of *Bacillus velezensis* SH-1471 genome with *Bacillus* strains 168, 3610, DSM7, FZB42, and SQR9 blast. **(9)** Mapping of Gene cluster for secondary metabolite synthesis of *Bacillus velezensis* SH-1471; Red: based on anti SMASH; Blue: based on BAGEL. BRIG software is used for SH-1471 loop mapping and comparative genome analysis.

The sequencing results of the whole genome of *B. velezensis* SH-1471 showed that it had a chromosome with a size of 4.15 Mb and a length of 4,181,346 bp. The GC-content was 46.18%, encoding 4187 genes. The gene coding rate was 88.63%, the longest sequence length was 17,103 bp, the shortest sequence length was 51 bp, the average length of coding genes was 885.06 bp, there were 4 plasmids, containing 136 Tandem repeat (TR) sequences, and the total length was 10,925 bp, the size is 6–282 bp, including 98 microsatellite DNA with a total length of 7148 bp and a size of 10–60 bp. There are 3 microsatellite DNA with a total length of 214 bp, containing 91 tRNA genes, 10 5S rRNA, 10 16S rRNA, and 23 S rRNA, and 86 ncRNAs. The genome sequencing data of *B. velezensis* SH-1471 was submitted to GenBank with Gene Bank accession CP128559. Seven prophages were predicted in chromosome sequence. 32 gene islands were predicted, and 251 protein coding genes in chromosome sequence contained signal peptide sequences. There is at least one or more transmembrane helical regions among the 1113 protein coding genes in the chromosome sequence. There are 122 protein coding genes in the chromosome sequence that contain secretory protein sequences. The genome map of *B. velezensis* SH-1471 and its comparative analysis with other *Bacillus* genomes are shown in Figs. 5, 6 and Table 2, in which the secondary metabolite Gene cluster based on anti SMASH and BRIG database analysis is also annotated.

**Table 2:** Genomic features of the *Bacillus velezensis* SH-1471 and related members of the *Bacillus* genus.

Features	*B. velezensis* SH-1471	*B. velezensis* SQR9	*B. velezensis* FZB42	*B. amyloliquefaciens* DSM7	*B. subtilis* 168
Genome size (bp)	4,181,346	4,117,023	3,918,589	3,980,199	4,214,630
G + C content (mol %)	46.18%	46.1%	46.4%	46.1%	43.5%
Protein-coding sequences	4187	4078	3693	3921	4106
Average CDS size (bp)	885	916	933	888	895
Percent of coding region	88.63%	90.7%	88%	87%	87%
Plasmid	4	0	0	0	0
Number of tRNAs	91	72	89	94	86
Ribosomal RNA operons	10	7	10	10	10
Phage-associated genes	7	218	44	185	268
GenBank sequence	CP128559.1	CP006890.1	CP000560.1	FN597644.1	AL009126.3

### Genome annotation results of strain SH-1471

**Figure 7:**
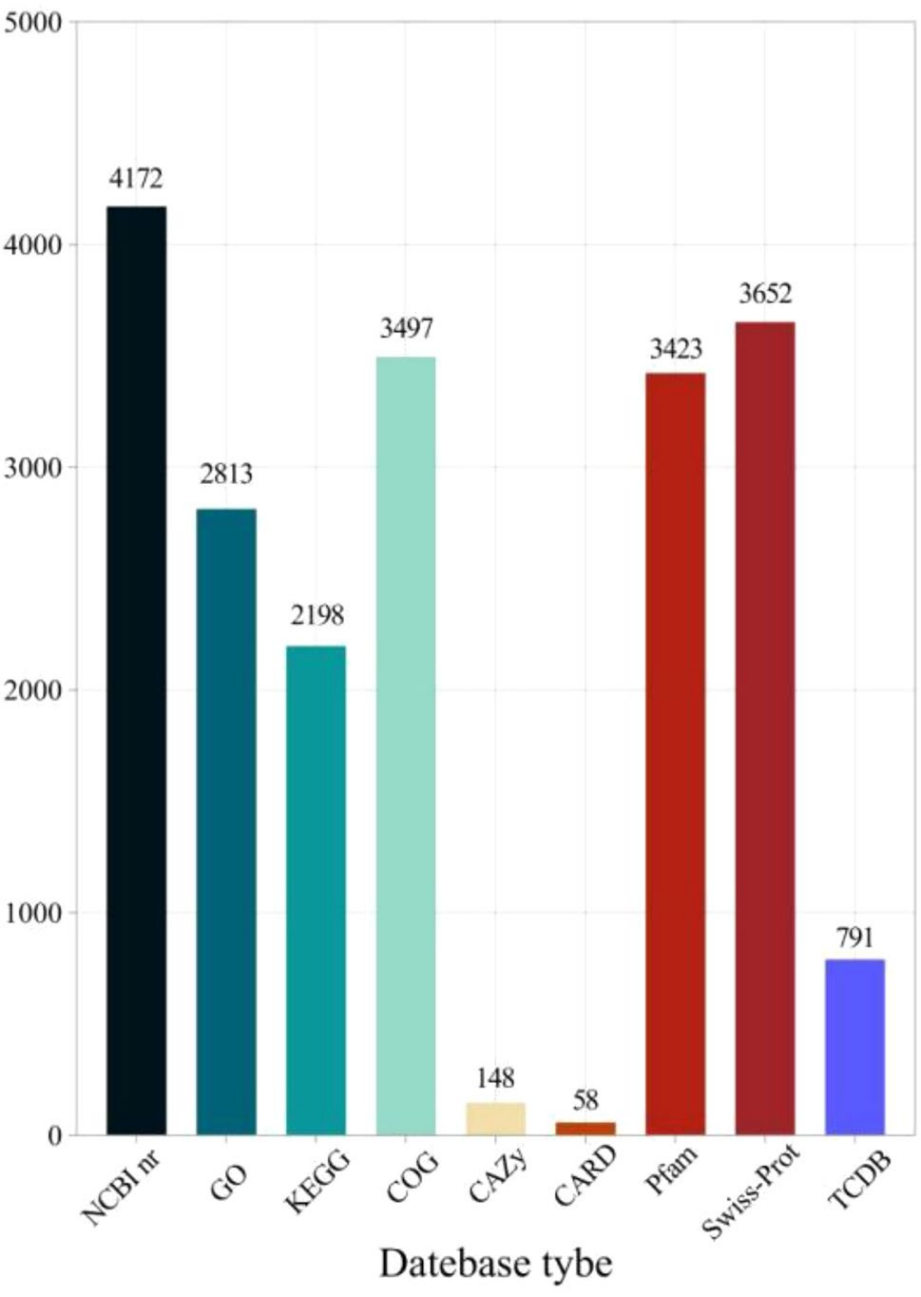
Distribution of gene function annotation database of *Bacillus velezensis* SH-1471. The X axis represents the names of each database, and the data in the figure indicates the total number of genes annotated by each database.

We used NCBI nr, eggNOG, KEGG, Swiss Prot, GO, TCDB, Pfam, *CAZy*, and CARD databases to perform a diamond comparison (E value ≤ 1e−5) of the predicted gene protein sequences with various functional databases, and selected the highest score comparison result (default identity ≥ 40%, coverage ≥ 40%) for annotation. The final annotated statistical data is shown in Fig. 7. In the NCBI nr, Swiss Prot, COG, Pfam, GO, and KEGG databases, there are many genes with functional annotations, namely 4172, 3652, 3497, 3423, 2813, and 2198, accounting for 99.64%, 87.22%, 83.52%, 81.75%, 67.19%, and 52.51% of the total genes; In the *CAZy* database, a total of 148 genes were annotated, accounting for 3.54%. In the TCDB database, a total of 791 genes were annotated, accounting for 18.89%. However, in the CARD database, the least annotated genes were obtained, accounting for 58, accounting for 1.39% of the total number of genes.

**Figure 8:**
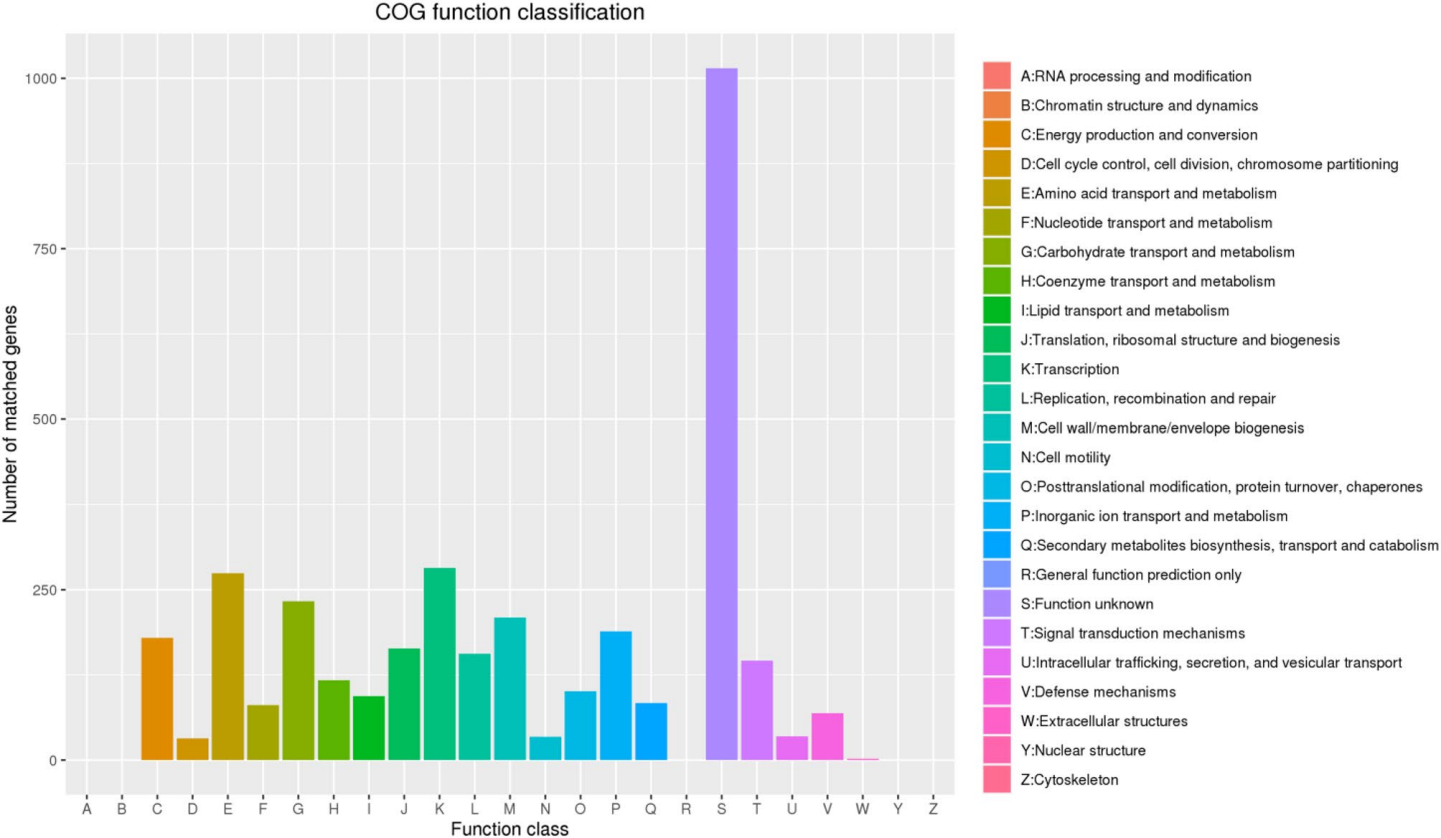
Functional annotation results of COG database of *Bacillus velezensis* SH-1471 genome. The COG functional annotations were divided into 26 categories. The COG categories are shown on the X-axis as alphabets, with category names on the right.

We used the COG database to annotate the protein of *B. velezensis* SH-1471. The classification results of 2875 genes annotated by COG on strain SH-1471 were shown in Fig. 7. Among them, there were 1015 genes with no clear function, which may be related to the lack of research on *B. velezensis* SH-1471 and the lack of reference genes. In addition, transcription annotation results were the most abundant, with a total of 282 genes, accounting for 8.09% of the total number of annotated genes. This is followed by amino acid transport and metabolism genes, with a total of 274 genes, accounting for 7.69% of the total number of annotated genes. The carbohydrate transport metabolism (233 genes, 6.54%), cell wall/membrane/envelope biogenesis (212 genes, 5.95%) and inorganic ion transport metabolism (189 genes, 5.31%) genes have also been more annotated, and 1047 genes (29.39%) have unknown functions that require further study in the future (Fig. 8).

**Figure 9:**
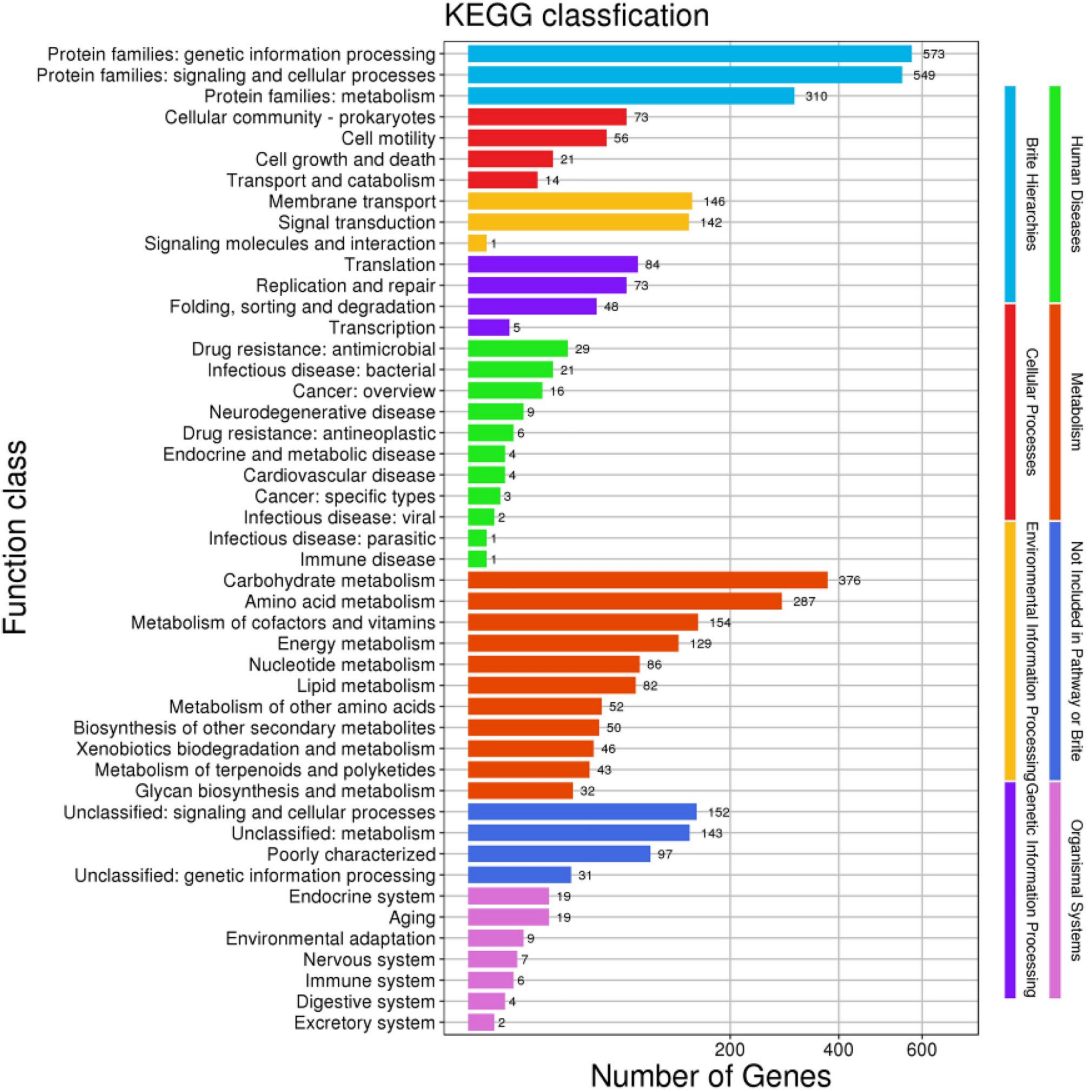
Functional annotation results of KEGG database of *Bacillus velezensis* SH-1471 genome. The KEGG orthologies were categorized into eight major categories: Brite Hierarchchies, Metabolism, Genetic Information Processing, Environmental Information Processing, Human Diseases, Not Included in Pathway or Brite, Cellular Processes and Organismal Systems.

The genome of *B. velezensis* SH-1471 has 2236 genes annotated in KEGG, divided into eight major categories and fifty subcategories. Among them, protein families: genetic information processing, protein families: signaling and cellular processes, and carbohydrate metabolism are the three most important metabolic pathways, with 573, 549, and 376 gene annotation results (Fig. 9).

**Figure 10:**
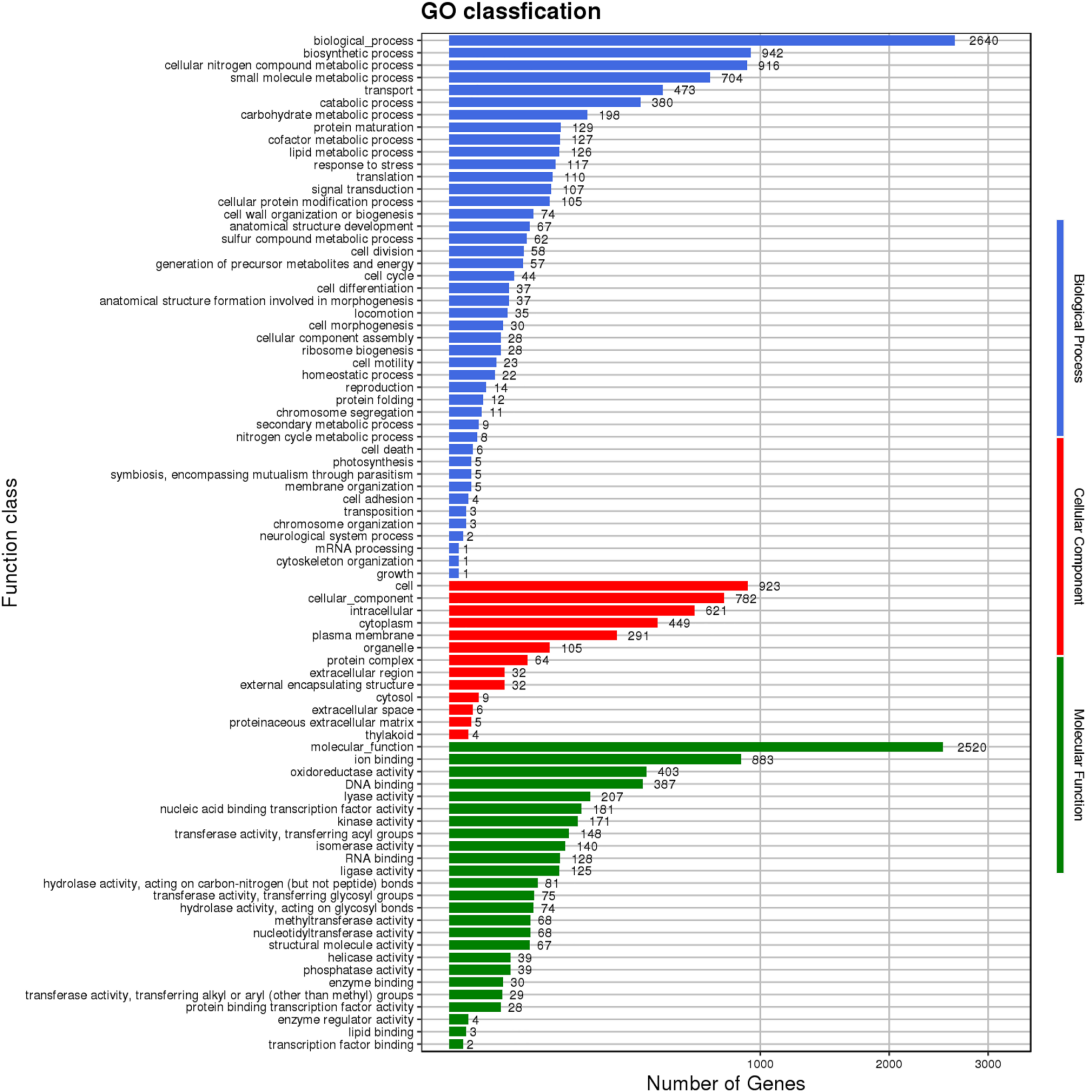
Functional annotation results of GO database of *Bacillus velezensis* SH-1471 genome. The GO assignments were divided into three categories (level 1) namely, biological process (red), cellular process (blue), and molecular function (green).

We compared and statistically analyzed the amino acid sequence of *B. velezensis* SH-1471 with the GO database to obtain the distribution of functional genes in the strain. A total of 2852 genes have been annotated in the GO database. The GO database annotates proteins based on three aspects: cellular component, biological process, and molecular function (Fig. 10). The biological process, cellular component, and molecular function branches each have 25, 11, and 10 branches, totaling 46 branches. A total of 3387 gene annotations were related to cell components, of which 923, 782 and 621 gene expression related to cells, cellar component and intelligent showed the highest correlation; A total of 7992 genes in the biological pathway category have been annotated, involving the most genes and biological pathways. There are 2640, 942, and 916 processes related to process, biological process, and cellular nitrogen compound metabolic process, respectively; A total of 6020 annotated results for molecular functional branches, consistent with molecular. The genes related to function, ion binding, and oxidoreductase activity are the most, with 2520, 883, and 403 genes, respectively.

### *CAZyme* gene families

**Figure 11:**
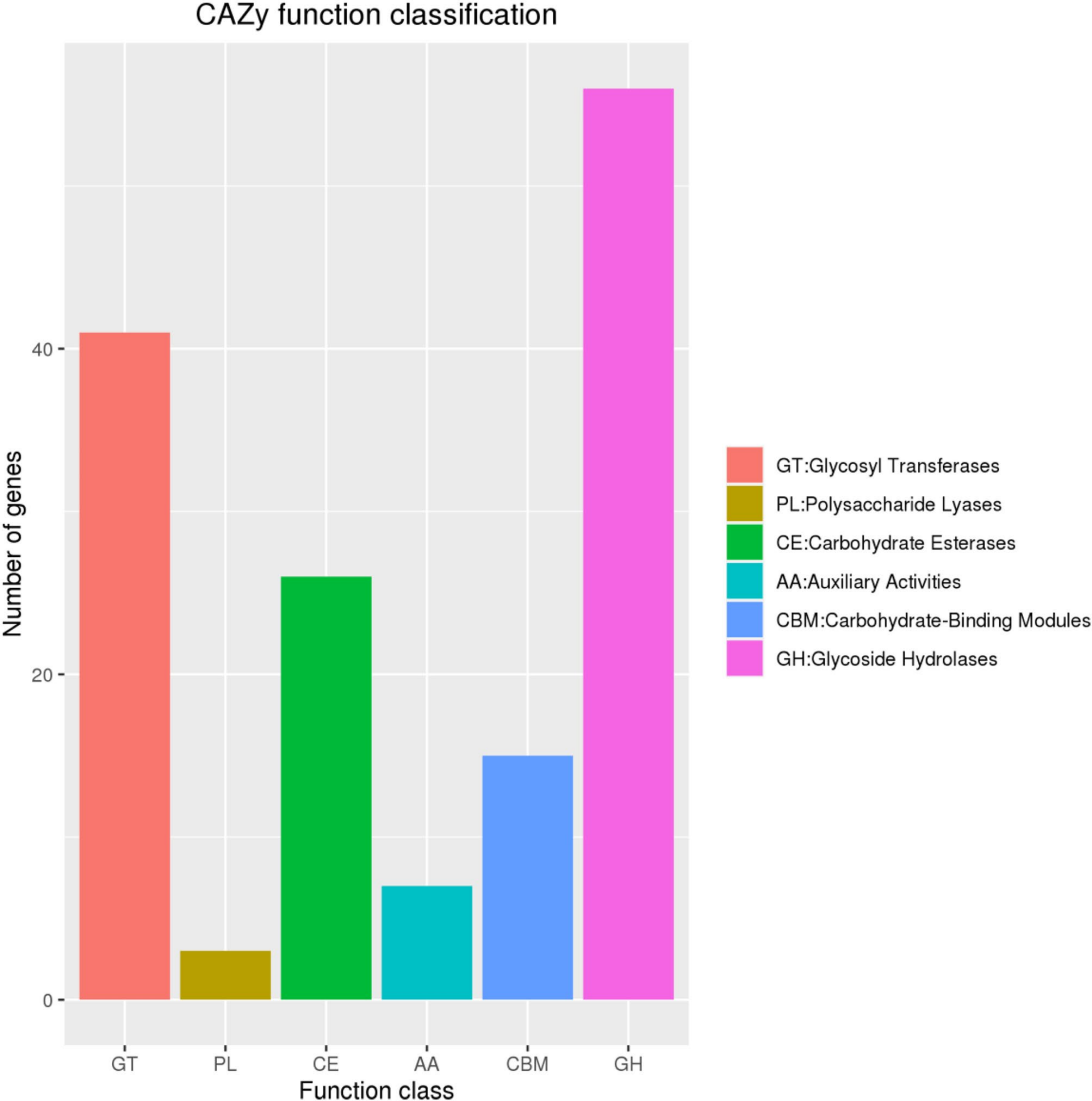
Functional annotation results of *CAZy* database of *Bacillus velezensis* SH-1471 genome. The X-axis is the abbreviation of each function of CAZy database, and the specific function of each abbreviation is on the right side of the figure.

We compared the genome sequence with the *CAZy* database and found that there were 148 genes encoding protein domain belonging to the *CAZy* family in the genome of *B. velezensis* SH-1471(Fig. 11), including 56 proteins of 20 Glycoside hydrolase (GHs) family, 41 proteins of 9 glycosyl transferases (GTs) family, 26 proteins of 6 carbohydrate esterases (CEs) family 3 types of polysaccharide-lyases (PLs), 7 auxiliary activities, and 15 types of carbohydrate binding modules (CBMs). We also found many genes in the genome of *B. velezensis* SH-1471, which are involved in coding endo glucan (endo-1,4-β-d-glucose) β-Glucosidase (β-Glossidase) and α-Amylase (*amyE*), which can decompose and utilize plant disease residues consisting of sugars and proteins in soil.

### Antibiotic resistance (CARD) analysis

We compared and analyzed the genome of *B. velezensis* SH-1471 with the CARD database, and found 64 antibiotic resistance genes in the genome of *B. velezensis* SH-1471, including 39 antibiotic resistance genes, 22 antibiotic target genes, and three antibiotic biosynthesis genes.

### Prediction of NP BGCs in the Genome of Strain SH-1471

**Figure 12:**
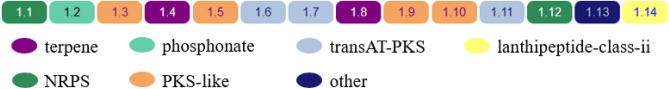
Location of secondary metabolite clusters in the genome of *Bacillus velezensis* SH-1471.

We used the anti SMASH database to predict that 14 secondary metabolite biosynthesis Gene cluster (Table 3, Fig. 12, supplementary Fig. S3) were found in the genome of *B. velezensis* SH-1471, including two terpenes, one phosphate, three transAT-PKS, one lanthipeptide class II, two NRPS and four PKS like. Among them, six clusters were identified to participate in the synthesis of surfactant, macroactin, bacillaene, difficidin, bacillus actin, and bacillus sin. In addition, the third Gene cluster is responsible for the synthesis of butyrosin A/butyrosin B, the seventh Gene cluster is responsible for the synthesis of fengmycin, and the 14th Gene cluster is responsible for the synthesis of mersacidin. We found that all predicted Gene cluster were related to bacteriostasis. We compared the secondary metabolite cluster of strain SH-1471 with the clusters of four Bacillus strains (FZB42, SQR9, DSM7, and 168), and the results showed that there may be potential new metabolites in the genome of strain SH-1471. For example, cluster 3 encodes PKS-like, which has a genetic similarity of 7% with butyrosin A/butyrosin B, but is inconsistent with the biosynthetic genes of butyrosin A in the MIBiG database. In addition, the phosphonate encoded by cluster 2 and the lanthipeptide class ii encoded by cluster 14 do not exist in the genomes of all four reference *Bacillus* strains. In addition, we annotated the aforementioned genes through the MIBIG database and annotated the annotation results with the highest BLAST score for each gene (Table 4). We also conducted BAGEL analysis on the *B. velezensis* SH-1471 genome and identified five different bacteriocins and RiPP clusters (Table 5).

**Table 3:** Gene clusters of secondary metabolites of *Bacillus velezensis* SH-1471.

*Bacillus velezensis* SH-1471					Secondary metabolites clusters in *Bacillus* strains			
Cluster ID	bp	Gene number	Cluster type	Most similar known cluster (% of Genes show similarity)	FZB42	SQR9	DSM7	168
1	54,288	28	NRPS	Surfactin (82%)	+	+	+	+
2	12,142	6	Phosphonate		−	−	−	−
3	41,245	21	PKS-like	Butirosin A/butirosin B (7%)	−	−	−	−
4	16,579	6	Terpene		+	+	+	+
5	87,831	21	TransAT-PKS	Macrolactin H (100%)	+	+	−	−
6	100,286	25	TransAT-PKS, T3PKS, NRPS	Bacillaene (100%)	+	+	+	+
7	134,678	45	NRPS, transAT-PKS, betalactone	Fengycin (100%)	+	+	+	+
8	23,884	7	Terpene		+	+	+	+
9	41,101	8	T3PKS		+	+	+	−
10	93,787	31	TransAT-PKS	Difficidin (100%)	+	+	−	−
11	51,797	25	NRP-metallophore,NRPS,RiPP-like	Bacillibactin (100%)	+	+	+	+
12	68,421	13	NRPS		+	−	+	-
13	41,419	23	Other	Bacilysin (100%)	+	+	+	+
14	23,189	15	Lanthipeptide-class-II	Mersacidin (100%)	−	−	−	−

**Table 4:** Annotation results of *Bacillus velezensis* SH-1471 secondary metabolic synthesis gene cluster based on MIBIG.

Query name	MIBIG cluster	BLAST score	Main product
chr_316	BGC0000433	6536	Surfactin
chr_634	BGC0000982	320.0	Cystothiazole A
chr_950	BGC0000693	641	Butirosin A
chr_1051	BGC0000172	571.0	Basiliskamide A
chr_1461	BGC0000181	7934	Macrolactin H
chr_1727	BGC0001089	10,576	Bacillaene
chr_1878	BGC0001095	3403	Fengycin
chr_1946	BGC0002137	636.0	Mutaxanthene A Mutaxanthene B Mutaxanthene C
chr_2017	BGC0001289	895.0	Penisin
chr_2331	BGC0000176	1454	Difficidin
chr_3145	BGC0001185	3382	Bacillibactin
chr_3479	BGC0000403	2776.0	Pelgipeptin
chr_3738	BGC0001184	929	Bacilysin
chr_3920	BGC0000527	2096.0	Mersacidin

**Table 5:** Predicted bacterial *Bacillus velezensis* SH-1471 and RIPP clusters based on BAGEL.

AOIOI	Start	End	Class
chr.0.AOI_01	3257423	3277615	266.1; Amylocyclicin
chr.0.AOI_02	298607	318742	132.2; LCI
chr.0.AOI_03	708455	728869	11.3; Colicin
chr.0.AOI_04	4019207	4043119	62.1; Mersacidin
chr.0.AOI_05	3207239	3227401	320.1; ComX3

In addition, we also used PRISM (https://prism.adapsyn.com/results/2333d5b064a84a481a05a15667198e9) algorithm to predict the structure of genetic coding natural product of *B. velezensis* SH-1471 genome. The results showed that there were 13 clusters of predicted compounds, including four NRPs, four PKs, one Class II/III bacteriocin, one ComX, one Bacilysin, one bacterial head-to-tail cyclized peptide, and 1 class II lantipeptide (Table 6, supplementary Fig S4).

**Table 6:** Predicted the type and quantity of secondary metabolites of *Bacillus velezensis* SH-1471 based on PRISM.

Clusters	Cluster type
Cluster 1	Class II/III confident bacteriocin
Cluster 2	Nonribosomal peptide
Cluster 3	Polyketide
Cluster 4	Polyketide, nonribosomal peptide
Cluster 5	Polyketide, nonribosomal peptide
Cluster 6	Nonribosomal peptide
Cluster 7	Polyketide
Cluster 8	ComX
Cluster 9	Nonribosomal peptide
Cluster 10	Bacterial head-to-tail cyclized peptide
Cluster 11	Nonribosomal peptide
Cluster 12	Bacilysin
Cluster 13	Class II lantipeptide

### Gene analysis for promoting plant growth and enhancing plant immunity

Based on the annotation results of eggNOG, Swiss Prot, and NCBI nr databases, we further screened for genes that promote plant growth and enhance plant immunity. The results showed that there are a series of genes related to plant root colonization and biofilm formation in the genome of *B. velezensis* SH-1471 (Supplementary Table S2), including *sacB*, *sacT*, *tasA*, and *tapA*. At the same time, we also found many genes coding for the synthesis of plant derived substrate enzymes, such as the genes *ganA*, *xynA* and *xynD* related to the coding of xylanase, the genes *bglC* and *XynC* related to the coding of glucan enzyme, and the gene *ganA* related to the encoding of galactose utilization enzyme (participating in the utilization of cellulose and hemicellulose in plant cell wall). In addition, it also has genes for synthesizing Indole-3-acetic acid, including *yhcX*, *dhaS* and *ysnE*, and genes for synthesizing acetyl and 2,3-butanediol, including *alsD*, *alsS* and *alsR*. Therefore, *B. velezensis* SH-1471 can promote plant growth and induce systemic resistance while having good biological control potential.

### Analysis of stress resistance genes

We searched for genes related to stress adaptation based on annotations of the *B. velezensis* SH-1471 genome in eggNOG, Swiss Prot, and NCBI nr databases. The results showed that there were many genes in the genome of *B. velezensis* SH-1471 that promoted strain adaptation to harsh stresses, including pH stress resistance (Supplementary Table S3), oxidative stress resistance (Supplementary Table S4), ion and heavy metal stress resistance (Supplementary Table S5), thermal stress resistance (Supplementary Table S6) and other stress resistance (Supplementary Table S7).

### Analysis of drug resistance genes

In addition, we searched for genes associated with drug resistance based on annotation results of the *B. velezensis* SH-1471 genome. The results showed that there were 102 resistance-related genes in the genome of strain SH-1471 (Supplementary Table S8).

### Untargeted metabolomics analysis of strain SH-1471

We used untargeted metabolomics to detect and analyze the fermentation broth of *B. velezensis* SH-1471, and identified and quantified a total of 482 identifiable metabolites, including 298 metabolites based on BioDeepDB database alignment, 72 metabolites based on MetaDNA database alignment, 94 metabolites based on MoNA database alignment, and 18 metabolites based on mzCloud database alignment. Among these recognizable compounds, carboxylic acids and their derivatives have the highest number, accounting for 14.45% of all metabolites, followed by fatty acyl groups (10.98%), benzene and substituted derivatives (6.55%) and benzene and organic oxygen compounds (5.39%), steroids and steroid derivatives (5.39%) and indole and its derivatives (2.71%). In addition, we compared strain metabolites to the KEGG database, with 28 for metabolism of terpenoids and polyketides and 69 for Biosynthesis of other secondary metabolites (Supplementary Fig S5). Among the metabolites of the metabolism of terpenoids and polyketides, costunolide and capsidiol are directly related to bacteriostatic (Supplementary Fig S6); Among the biosynthesis of other secondary metabolites, gingerol, luteolin, genistein, tabtoxinine-beta-lactam, (2S)-Liquiritigenin, and D-phenyllactic acid are directly associated with bacteriostatic properties (Supplementary Fig S7).

### Potting test results of SH-1471

After 30 days, the disease index, incidence rate and agronomic characters of tomato seedlings under each treatment were determined. Results As shown in Supplementary Table S9 and Supplementary Fig S8, after 30 days of inoculation, SH-1471 could effectively inhibit the occurrence of tomato wilt disease, and the disease index was as high as 79.8 in the control treatment with only the pathogen (CK1). The disease index of tomato seedlings treated with the fermentation solution of strain SH-1471 was 2.2, and the control effect was 93.8%. In addition, strain SH-1471 also had a certain growth-promoting effect on the growth of tomato seedlings. Compared with the control group, the plant height, stem circumference, root length, root weight, fresh weight and dry weight of above ground parts of tomato seedlings were significantly improved after treatment with strain fermentation solution.

## Discussion

*B. velezensis* can secrete a variety of secondary metabolites to inhibit plant pathogens, which has a wide range of applications in agriculture and is one of the common biocontrol bacteria^15^. Numerous studies have shown that strain sequencing by the 16S rRNA gene sequence alone is not effective in distinguishing *B. velezensis* from its peers. Therefore, we determined the classification location of strain SH-1471 based on multisite gene sequence analysis (MLSA) of three housekeeping genes (16S rRNA, *gyrA* and *gyrB*), and the results showed that strain SH-1471 was closely related to *B. velezensis* BCRC 17467, so the strain was identified as *B. velezensis*. In this paper, the whole genome size of *B. velezensis* SH-1471 was found to be 4,181,346 bp by whole genome sequencing and analysis of *B. velezensis* SH-1471. The genome sizes of the more classical *B. velezensis* FZB42, LS69 and SQR9 are 3,918,596 bp, 3,917,761 bp and 4,117,023 bp, respectively. The G + C content was a feature of microbial taxonomic description, with 46.2% G + C content in the genome of *B. velezensis* SH-1471 and 46.4%, 46.4% and 46.1% in FZB42, LS69 and SQR9, respectively. The *B. velezensis* SH-1471 genome contains 4187 protein-coding genes, which are functionally annotated through databases such as NCBI nr, eggNOG, KEGG, Swiss-Prot, GO, TCDB, Pfam, *CAZy* and CARD. We found that 4172 genes in the genome of *B. velezensis* SH-1471 were annotated on the NCBI nr database, similar to the identification results, and the amplified sequence of *B. velezensis* SH-1471 was most similar to that of *B. velezensis*.

In addition, the prediction of *CAZy* in the *B. velezensis* SH-1471 genome found that the highest content was 56 gene glycosidic hydrolases (GHs), followed by 41 gene glycosidyl transferase family proteins (GTs) and 26 gene carbohydrate esterases (CEs), and also had 3 genes of polysaccharide lyases (PLs), which can degrade cellulose and hemicellulose, chitin, starch, xylan and peptidoglycan^16^, while the cell walls of most pathogenic fungi are mainly composed of cellulose, dextran, and chitin^17^, similarly, we found that strain SH-1471 had cellulose-degrading activity in functional assays. In addition, GTs are important for surface structures recognized by the host immune system^18^. Thus, it was shown that *B. velezensis* SH-1471 has the potential to resist pathogens and immune stimulation.

We found that *B. velezensis* SH-1471 had a good inhibitory effect on eight pathogenic microorganisms, including *S. scrotiorum*, *P. mateuciicola*, and *F. oxysporum*, and it also has a good control effect on tomato wilt. The above results indicated that this strain has great application potential in biological control of plant diseases. Previous studies have shown that *Bacillus* can produce various substances with broad-spectrum antibacterial activity, including lipopeptide antibiotics, bacteriocins and antibacterial proteins, and the means of genomics are an effective means to mine the functional gene clusters of the strain and analyze the antagonistic mechanism of the strain to pathogenic microorganisms^19–22^. We annotated the *B. velezensis* SH-1471 genome by Anti-SMASH and found that there were 14 bacteriostatic active substance synthesis gene clusters in the secondary metabolite biosynthesis gene cluster in the *B. velezensis* SH-1471 genome (Table 2). We found substances such as chr_3479 pelgipeptin, chr_950 noted butirosin A, and chr_3920 annotated as mersacidin, and these substances have a wide range of antimicrobial activity against gram-negative and gram-positive bacteria. In addition, we performed BAGEL predictions on the *B. velezensis* SH-1471 genome, which yielded 5 different bacteriocin and RiPP clusters, including amylocyclicin, LCI, colicin, mersacidin, and ComX3. Related studies have shown that amylocyclicin has high antimicrobial activity against gram-positive bacteria^23^, and mersacidin has extremely high inhibitory activity against methicillin-resistant *Staphylococcus aureus*^24^. We also predicted the structure of genetically encoded natural products in the genome of *B. velezensis* SH-1471 by the PRISM algorithm.

We compared the annotations of various databases and found that in addition to a variety of secondary metabolites that produce antibacterial or antifungal activity, we found that the *B. velezensis* SH-1471 genome contains 72 genes that promote plant growth and improve plant immunity, including a series of genes related to root colonization and biofilm formation, including *sacT*, *sacB*, *spo0A* and *CapD*, which are associated with biological control^25–27^, in addition, there are polysaccharide biosynthesis proteins *yfnF* and *CapD*^28^. In addition, we also found through indoor experiments that SH-1471 has extremely strong biofilm synthesis ability, which will be more conducive to promoting SH-1471's colonization in plant roots .There are also genes such as *xynB* and *xynC* (encoding xylanase)^29^, *bglC* and *XynC* (encoding glucanase)^30^, and *ganA-lacR* operons (encoding enzymes for galactose utilization)^31^, which encode enzymes that utilize substrates of plant origin. So as to promote plants to use cellulose and hemicellulose in cell wall. Our results show that *B. velezensis* SH-1471 has *swrAA*, *swrB* genes, which encode cluster kinesin^32^, exopolysaccharide operons (*epsA-O*) associated with capsular biosynthesis^21^, as well as many genes involved in flagellar biosynthesis, such as *fliD*, *flgK*, and *hag*, are thought to enhance cluster motility and colonization^33–35^, as well as *SinR* and its antagonist *SinI*, which are pleiotropic DNA-binding proteins that are necessary for spore production and subtilisin synthesis^36,37^. We found that *B. velezensis* SH-1471 can colonize a large number of plant roots, which is beneficial for *B. velezensis* SH-1471 plays a long-term role in soil. It also has a gene encoding the *TasA* protein, which binds cells together in biofilms and is implicated in spore production^38^. In addition, *B. velezensis* SH-1471 also has a variety of genes encoding 3-hydroxy-2-butanone synthesis-related proteins, including acetolactate decarboxylase (*alsD*), acetolactate synthase (*alsS*), transcriptional regulator (*alsR*), and 2,3-butanediol dehydrogenase (*bdhA*), a compound that has been reported to improve plant growth and trigger systemic resistance^4,39^. In addition, *B. velezensis* SH-1471 contains genes required to synthesize indole-3-acetic acid (*yhcX*, *dhaS* and *ysnE*), acetoin, and 2,3-butanediol (*alsD*, *alsR*, *alsS* and *pta*)^40,41^. We have also verified this fact through growth promoting experiments, and our experimental results indicate that *B. velezensis* SH-1471 can significantly promote root development in plants. We found that strain SH-1471 has the functions of dissolving inorganic phosphorus and nitrogen fixation, which can promote the degradation of insoluble compounds in soil, increase the content of available phosphorus and soluble nitrogen in soil, promote the germination rate of crops and promote the growth of crop roots, and play an important role in improving soil fertility and the utilization rate of phosphate fertilizer. The siderophore synthesis ability of strain SH-1471 is also an important way to induce plant system resistance to resist pathogenic bacteria, and can promote plant growth and induce systemic disease resistance by secreting siderophores to promote plant root phylogenetic development and nutrient uptake, and induce systemic disease resistance.

*Bacillus* are widely used as biological control agents because they are extremely resistant to adverse environments such as heat, pressure and salinity^15^. Our analysis of the harsh environment resistance gene in the genome of *B. velezensis* SH-1471 found that the genome of the strain contains a large number of genes that help the strain adapt to harsh conditions, such as 5 F1F0 ATPases, 11 Na( +)/H( +) anti-transporters, 9 cation/H( +) antitransportases, and multiple proton ATPases and related subunits. Previous studies have shown that F1F0-ATPase, cationic/H( +) antiporter and Na( +)/H( +) antiporter have the effect of exporting protons from the cytoplasm, which is considered to be the main factor in regulating cell pH and increasing the resistance of strains to acid^42,43^. In addition, we identified genes associated with oxidative stress resistance, such as superoxide dismutase, in the *B. velezensis* SH-1471 genome. Magnesium transporters, zinc transporters, and metal-resistant proteins for ionic and heavy metal stress resistance were identified in the *B. velezensis* SH-1471 genome. A number of heat shock proteins were also identified, indicating the heat tolerance of *B. velezensis* SH-1471^44^. In addition, we have included genes encoding general stress response proteins, DNA repair proteins, cell wall integrity, and stress response components in the *B. velezensis* SH-1471 genome that help the strain cope with harsh environments^45,46^.

We also analyzed the resistance gene for *B. velezensis* SH-1471. According to the annotation results of protein-coding genes, 102 genes associated with drug resistance, including ABC multidrug transporter, multidrug resistance protein, MFS transporter, MFS multidrug transporter and MFS reverse transporter (Supplement Table S7), related studies have shown that ABC multidrug transporter may increase resistance to azoles^47^. The MFS transporter family is a multidrug efflux system that transports a variety of structurally independent compounds from cells, including cycloheximide and azoles, making the strain resistant to many compounds^48^. In the soil environment, there are not only a large number of pathogenic fungus and bacteria, but also a large number of closely related functional strains. However, the nutrient content in the rhizosphere of plants is limited, and functional bacteria need to compete with other microorganisms besides pathogenic bacteria for nutrients. Therefore, functional bacteria should have strong rhizosphere competitiveness. Previous studies have found that *B. velezensis* FZB42 can secrete plantazolin and amyocyclin to inhibit the growth of some *Bacillus* spp. In *B. velezensis* SQR9, it also encodes multiple novel antibacterial fatty acids to inhibit the growth of other *Bacillus* spp^49^. In the results of this study, we found that *B. velezensis* SH-1471 can also inhibit the growth of various closely related strains of *Bacillus*. Our experimental results indicate that it has strong competitiveness among close source strains of *Bacillus*.

In conclusion, our test and analysis results show that *B. velezensis* SH-1471 has good potential in biological control and plant growth, in addition, genomic information of *B. velezensis* SH-1471 will help reveal the molecular mechanism of its antimicrobial activity.

## Conclusion

The strain SH-1471, isolated from healthy tobacco rhizosphere soil, had strong antagonistic activity against a variety of plant pathogens, and it also had a good control effect on tomato wilt, could be colonized in a variety of plant roots and promote plant growth, and had the ability to produce proteases, cellulase, dissolve inorganic phosphorus, nitrogen fixation, and siderophore. The above results indicated that it has broad application potential in biological control of plant diseases and the promotion of crop growth. Combining whole genome sequencing and multilocus gene sequence analysis (MLSA) confirmed that the strain belonged to *B. velezensis*.

The *B. velezensis* SH-1471 gene containers 14 gene clusters of metamaterials of this organism that have been shown to have antimicrobial activity. In addition, *B. velezensis* SH-1471 genome contains some genes related to bacterial colonization and plant growth, as well as a large number of genes related to resistance to environmental stresses, including pH stress, heat stress, oxidative stress, ionic and heavy metal stress. The above results suggest that *B. velezensis* SH-1471 may be a promising biocontrol strain for plant diseases, and the results of this study will contribute to further understanding of the biological control mechanism of *B. velezensis*.

## Material and methods

### Source of strain

*B. velezensis* SH-1471 was isolated and preserved in the rhizosphere soil of healthy tobacco by the Institute of Agricultural Environmental Resources, Agricultural science, Yunnan Province (Deposit No.: CCTCC No: M 2022923, Patent No.: ZL 2022 1 1479280.X).

The indicator pathogens and *Bacillus* involved in this paper were isolated, identified and preserved by the Institute of Agricultural Environmental Resources of the Agricultural science of Yunnan Province, such as *F. oxysporum*, *A. alternate*, *E. turcicum*, *P. matteucicola*, *D. ere*s, *C. microtianae*, *P. parasitica* and *S. sclerotiorum.*

### Culture medium

NA medium (peptone 10 g/L, beef powder 3 g/L, NaCl 5 g/L, pH 7.0, agar 18 g/L), NB medium (peptone 10 g/L, beef powder 3 g/L, NaCl 5 g/L, pH 7.0), PDB medium (potato 200 g/L, glucose 20 g/L, pH 7.0), PDA medium (potato 200 g/L, glucose 20 g/L, agar 15-20 g/L, pH 7.0), LB medium (10 g/L tryptophan, 5 g/L yeast extract, 10 g/L NaCl), MSgg medium (100 mM 3-(N-morpholino) propane sulfonic acid (MOPS), 5 mM potassium phosphate, 2 mM MgCl_2_, 700 μM CaCl_2_, 50 μM MnCl_2_, 50 μM FeCl_3_, 1 μM ZnCl_2_, 2 μM thiamine, 0.5% (w/v) glycerol, 0.5% (w/v) glutamate, 50 μg/mL tryptophan, 50 μg/mL phenylalanine, and 50 μg/mL threonine, pH 7.0). In addition, we used inorganic phosphorus selection media, casein medium, cellulose medium, amylase screening medium, CAS bilayer plates to determine the diverse biological activity of SH-1471^14^.

### Antagonistic test of plant pathogenic fungi

Plate confrontation experiment: *F. oxysporum* was used as an indicator pathogen. A 3 mm pathogen cake was inoculated in the center of PDA medium, and functional strains were inoculated in a cross shaped manner at a distance of 25 mm from it. The uninoculated plate was used as a control, with three replicates for each strain. The bacteria were incubated in a constant temperature incubator at 25–28 ℃ for 5–7 days under dark conditions, and the antibacterial rate was calculated. In addition, SH-1471 and tomato fusarium wilt were cultured in a constant temperature incubator at 25 ℃, and the mycelia on the edge of the inhibition zone were selected for scanning electron microscopy after 15 days of treatment for three replicates.

$${\text{Inhibition rate }}\left( \% \right)\, = \,\left( {{\text{Colony diameter of the control group}} - {\text{colony diameter of the treatment group}}} \right)/\left( {{\text{colony diameter of the control group}} - 0.{3 } {\text{cm}}} \right)\, \times \,{1}00.$$

### Determination of biofilm formation and root colonization of strain SH-1471

This study used tomatoes, chili peppers, and cucumbers as experimental plants. The variety of cucumber is “fruit dry cucumber” (Hangcheng Seed Industry Co., Ltd, JiangXi, China); the pepper variety is “Huayu No.1 original pod pepper” (Xingyun Seed Industry Co., Ltd, HeBei, China); the tomato variety is “Maofen 802” (Yuyi Seed Industry Co., Ltd, XiAn, China), the above plant seeds are generously provided by Dr. Peiwen Yang (Yunnan Academy of Agricultural Sciences, Kunming, China).

The experimental research and field studies on plants (either cultivated or wild), including the collection of plant material, are comply with relevant institutional, national, and international guidelines and legislation. This study qualitatively measured the biofilm formation ability of the strain using MSgg culture medium^50^. The colonization abilities of strain SH-1471 on tomato, cucumber and pepper root were conducted according to previously reported methods^51^.

### Plant growth-promoting assay of strain SH-1471

Firstly, we disinfect the surface of tomato, pepper, and cucumber seeds and germinate them under sterile conditions, then transfer them to a square board covered with sterile filter paper. Then, we mixed the fermentation broth of strain SH-1471 with 1/5 Hoagland to obtain 0.5 × 106 cfu/mL of 1/5 Hoagrand solution, sterile 1/5 Hoagrand solution as control. Finally, we take 15 mL of each solution and place it in a light temperature chamber with a relative humidity of 70% for 12 h (26 ℃)/12 h in darkness (22 ℃). After 7 days of cultivation, we recorded the development of plant roots.

### Total DNA extraction and identification of strains

Inoculate strain SH-1471 in NB medium, incubate at 37 ℃ at 180 r/min for 48 h, centrifuge at 10,000 rpm/min, and use sterile 0.22 μL microporous membrane is used to filter the residual bacteria, and the bacterial genome DNA extraction kit is used to extract the genome. Refer to the kit instructions for the operation steps. In addition, we identified the taxonomic location of strain SH-1471 based on ANI (Average Nucleotide Identity) and dDDH (digital DNA-DNA Hybridization) analyses^52^ and multi site gene sequence analysis (MLSA) of three identified genes (16S rRNA, *gyrA*, and *gyrB*). Firstly, we use MAFFT(https://www.ebi.ac.uk/Tools/msa/mafft/) arrange and align gene sequences, then prune with MEGA7 to remove areas of unclear arrangement, and finally construct a phylogenetic tree using the maximum likelihood method in raxmlGUI.

### Genome sequencing and assembly

Using the Whole Genome Shotgun (WGS) strategy, a library of different inserted fragments was constructed, and the next-generation sequencing technology (NGS) was used based on the Illumina NovaSeq sequencing platform (2 × 150 bp end-to-end reads, while utilizing third-generation single molecule sequencing technology and using the PacBio Sequel sequencing platform to sequence these libraries separately. After sequencing, HiFiasm, Unicycler, Flye and other software were used to assemble and obtain the contig sequence. In addition, the high-quality data of the second generation were corrected by the software pilon to obtain the complete sequence^53^.

### Genomic annotation and analysis of strains

After obtaining the assembled genome, RepeatModeler (version 1.0.8) and RepeatMask (version 4.0.5) software were used to perform de novo prediction of repetitive sequences in the genome. When conducting non coding RNA analysis, tRNA was predicted using tRNAscan-SE^54^, and rRNA was predicted using Barrnap software. The predictions for other non coding RNAs were mainly obtained by comparing them with the Rfam database^55^.

We used GeneMarkS v^56^software to predict protein coding Gene prediction of the whole gene sequence; PhiSpy was used to predict the presence of Prophage in the genome^57^; IslandViewer 4 is used to predict the presence of gene islands in the genome^58^. We annotated the protein coding genes by searching KEGG databases^59^ and NCBI nr, eggNOG, Swiss Prot, GO, TCDB, Pfam, *CAZy*, CARD, etc. In addition, the genome annotation results of *B. velezensis* SH-1471 were used to search for genes related to stress adaptation, plant growth promotion, enhancing plant immunity, and drug resistance. Secreted proteins and membrane proteins were predicted using SingalP and TMHMM.

### Cluster prediction of secondary metabolite synthesis genes

We used the method combining anti SMASH 6^60^ with ClusterBlast, ActiveSiteFinder, Cluster PFam analysis, SubClusterBlast and PRISM 4 to identify and compare the secondary metabolite BGCs in the genomes of *B. velezensis* SH-1471, *B. velezensis* FZB42, *B. velezensis* SQR9, *B. amyloliquefaciens* DSM 7, and *B. subtilis* 168. We also utilized BAGEL 4^60^ to mine RiPPs and bacteriocins in BGCs, and PRISM 4 to predict the structure of secondary metabolites in strains^61^. In these database systems, including Hidden Markov model (HMM) principle^62^, BLAST algorithm^63^, PFAM^34^, GenBank^64^, UniprotKB^65^, bactibase^66^, CAMPR3^67^ and MiBig database^68^ are used for BGC annotation. In addition, NapDos has also been used to search for KS and C domains in these genomic sequences^69^.

### Untargeted metabolomics analysis of metabolites in strains

We used NB culture medium to prepare strain fermentation broth, and the prepared fermentation filtrate sample was sent to Shanghai Parsenor for untargeted metabolomics analysis. The test conditions were as follows: chromatographic conditions: the sample was separated using the Agilent 1290 Infinity LC ultra high performance liquid chromatography system (UHPLC) HILIC column; Column temperature 25 ℃; Flow rate 0.3 mL/min; Injection volume 2 μL; During the entire analysis process, the sample was placed in a 4 ℃ automatic sampler. Q-TOF mass spectrometry conditions: the samples are detected and analyzed respectively in the positive and negative ion modes of electrospray ionization (ESI). The sample was separated by UHPLC and subjected to mass spectrometry analysis using the Triple TOF 5600/6600 mass spectrometer (ABSCIEX).

### Prevention and control effect of tomato wilt and growth promotion of tomato seedlings

After the pathogens were cultured at 28 °C and 180 r/min for 5–7 days in PDB medium, they were diluted into 1.5 × 10^7^ CFU/mL spore suspension. The strain SH-1471 was cultured for 20–24 h and diluted into a 2 × 10^8^ CFU/mL bacterial suspension. The bacterial solution was inoculated by the root irrigation method, and the hole was pierced at about 3 cm of the rhizome of tomato seedlings with a glass rod, about 5 cm deep, 100 mL of pathogenic bacteria suspension per plant, and the same amount of functional strain suspension was added after 3 days of colonization of pathogenic bacteria, 10 strains per treatment, 3 replicates. CK1 (sterilized medium) and CK2 (pathogenic bacteria + sterilized medium) were used as controls, and the incidence was observed and recorded after 30 days, and the plant height, stem circumference, root length, root weight, aerial fresh weight and aboveground dry weight of tomato plants were measured at the same time.

The potted plants were cultured at constant temperature for 30 days, and the plant growth and incidence were observed and recorded according to the classification standard of tomato wilt^70^.

### Supplementary Information

Supplementary Information.

## Data Availability

The assembled genome sequences for strain SH-1471 were uploaded to the NCBI GenBank, accession number CP128559.

## References

[1] Coninck BD, Timmermans P, Vos C, Cammue PA, Kazan K (2015). What lies beneath: Belowground defense strategies in plants. Trends Plant Sci..

[2] Ongena M, Jacques P (2008). *Bacillus lipopeptides*: Versatile weapons for plant disease biocontrol. Trends Microbiol..

[3] Lim SBY (2018). Genome sequence of *Bacillus velezensis* SGAir0473, isolated from tropical air collected in Singapore. Genome Announc..

[4] Ōmura S (2001). Genome sequence of an industrial microorganism *Streptomyces avermitilis*: Deducing the ability of producing secondary metabolites. PNAS..

[5] Abdellaziz L (2018). Lipopeptide biodiversity in antifungal *Bacillus* strains isolated from Algeria. Arch. Microbiol..

[6] Chen XH (2009). Difficidin and bacilysin produced by plant-associated *Bacillus amyloliquefaciens* are efficient in controlling fire blight disease. J. Biotechnol..

[7] Kim SY, Lee SY, Weon HY, Sang MK, Song J (2017). Complete genome sequence of *Bacillus velezensis* M75, a biocontrol agent against fungal plant pathogens, isolated from cotton waste. J. Biotechnol..

[8] Santner A, Calderon-Villalobos L, Estelle M (2009). Plant hormones are versatile chemical regulators of plant growth. Nat. Chem. Biol..

[9] Bochow, H., El-Sayed, S.F., Junge, H., Stavropoulou A. & Schmiedeknecht G. Use of *Bacillus subtilis* as biocontrol agent. IV. Salt-stress tolerance induction by *Bacillus subtilis* FZB24 seed treatment in tropical vegetable field crops, and its mode of action/Die Verwendung von *Bacillus subtilis* zur biologischen Bekämpfung. IV. Induktion einer Salzstress-Toleranz durch Applikation von *Bacillus subtilis* FZB24 bei tropischem Feldgemüse und sein Wirkungsmechanismus. *J. Plant Dis. Protect*. 21–30. https://www.jstor.org/stable/43215378 (2001).

[10] Cui LX, Yang CD, Wei LJ, Li TH, Chen XY (2019). Isolation and identification of an endophytic bacteria *Bacillus velezensis* 8–4 exhibiting biocontrol activity against potato scab. Biol. Control.

[11] Sun PP, Cui JC, Jia XH, Wang WH (2017). Complete genome sequence of *Bacillus velezensis* L-1, which has antagonistic activity against pear diseases. Genome Announc..

[12] Domka J, Lee J, Bansal T, Wood TK (2007). Temporal gene-expression in *Escherichia coli* K-12 biofilms. Environ. Microbiol..

[13] Grossman AD, Lewis T, Levin N, Vivo RD (1992). Suppressors of a *spo0A* missense mutation and their effects on sporulation in *Bacillus subtilis*. Biochimie..

[14] Shen YX (2023). Isolation, identification and bio-activity of three *Bacillus* strains with biocontrol function. Biol. Bull..

[15] Glaeser SP, Kämpfer P (2015). Multilocus sequence analysis (MLSA) in prokaryotic taxonomy. Syst. Appl. Microbiol..

[16] Walker BJ (2014). Pilon: An integrated tool for comprehensive microbial variant detection and genome assembly improvement. PloS one..

[17] Lowe TM, Eddy SR (1997). tRNAscan-SE: A program for improved detection of transfer RNA genes in genomic sequence. Nucleic Acids Res..

[18] Kalvari I (2018). Rfam 13.0: Shifting to a genome-centric resource for non-coding RNA families. Nucleic Acids Res..

[19] Besemer J, Lomsadze A, Borodovsky M (2001). GeneMarkS: A self-training method for prediction of gene starts in microbial genomes. Implications for finding sequence motifs in regulatory regions. Nucleic Acids Res..

[20] Zhou Y, Liang YJ, Lynch KH, Dennis JJ, Wishart DS (2011). PHAST: A fast phage search tool. Nucleic Acids Res..

[21] Bertelli C (2017). IslandViewer 4: Expanded prediction of genomic islands for larger-scale datasets. Nucleic Acids Res..

[22] Blin K (2021). antiSMASH 6.0: Improving cluster detection and comparison capabilities. Nucleic Acids Res..

[23] Machado H, Sonnenschein EC, Melchiorsen J, Gram L (2015). Genome mining reveals unlocked bioactive potential of marine Gram-negative bacteria. BMC Genomics..

[24] Churchill GA (1989). Stochastic models for heterogeneous DNA sequences. Bull. Math. Biol..

[25] Altschul SF, Gish W, Miller W, Myers EW, Lipman DJ (1990). Basic local alignment search tool. J. Mol. Biol..

[26] Finn RD (2014). Pfam: The protein families database. Nucleic Acids Res..

[27] Benson DA (2012). GenBank. Nucleic Acids Res..

[28] UniProt Consortium (2015). UniProt: A hub for protein information. Nucleic Acids Res..

[29] Hammami R, Zouhir A, Lay CL, Hamida JB, Fliss I (2010). BACTIBASE second release: A database and tool platform for bacteriocin characterization. BMC Microbiol..

[30] Waghu FH, Barai RS, Gurung P, Idicula-Thomas S (2016). CAMP_R3_: A database on sequences, structures and signatures of antimicrobial peptides. Nucleic Acids Res..

[31] Medema MH (2015). Minimum information about a biosynthetic gene cluster. Nat. Chem. Biol..

[32] Ziemert N (2012). The natural product domain seeker NaPDoS: A phylogeny based bioinformatic tool to classify secondary metabolite gene diversity. PloS one..

[33] Xu S (2020). Whole-genome analysis of *Bacillus velezensis* ZF2, a biocontrol agent that protects cucumis sativus against corynespora leaf spot diseases. 3 Biotech..

[34] Liu DM, Huang YY, Liang MH (2022). Analysis of the probiotic characteristics and adaptability of *Lactiplantibacillus plantarum* DMDL 9010 to gastrointestinal environment by complete genome sequencing and corresponding phenotypes. LWT..

[35] Chen XH (2007). Comparative analysis of the complete genome sequence of the plant growth–promoting bacterium *Bacillus amyloliquefaciens* FZB42. Nat. Biotechnol..

[36] He P (2023). Structural mechanism of a dual-functional enzyme *DgpA/B/C* as both a C-glycoside cleaving enzyme and an O-to C-glycoside isomerase. Acta Pharmacol. Sin..

[37] Zhang J (2022). Characterization and antibacterial modes of action of bacteriocins from *Bacillus coagulans* CGMCC 9951 against Listeria monocytogenes. LWT..

[38] Appleyard AN (2009). Dissecting structural and functional diversity of the lantibiotic mersacidin. Chem. Biol..

[39] Rampelotto PH (2010). Resistance of microorganisms to extreme environmental conditions and its contribution to astrobiology. Sustain..

[40] Guo D (2020). Biocontrol of tobacco black shank disease (*Phytophthora nicotianae*) by *Bacillus velezensis* Ba168. Pestic Biochem. Phys..

[41] Grady EN (2019). Characterization and complete genome analysis of the surfactin-producing, plant-protecting bacterium *Bacillus velezensis* 9D-6. BMC Microbiol..

[42] Wang T, Liu X, Wu MB, Ge S (2018). Molecular insights into the antifungal mechanism of bacilysin. J. Mol. Model..

[43] DiCandia MA (2022). Identification of functional *Spo0A* residues critical for sporulation in *Clostridioides difficile*. J. Mol. Biol..

[44] Li W (2014). Analysis of the *Staphylococcus aureus* capsule biosynthesis pathway in vitro: Characterization of the UDP-GlcNAc C6 dehydratases *CapD* and *CapE* and identification of enzyme inhibitors. Int. J. Med. Microbiol..

[45] Meng F (2023). *LsrR*-like protein responds to stress tolerance by regulating polysaccharide biosynthesis in *Lactiplantibacillus plantarum*. Int. J. Biol. Macromol..

[46] MacCabe AP, Fernández-Espinar MT, De Graaff LH, Visser J, Ramón D (1996). Identification, isolation and sequence of the *Aspergillus nidulans xlnC* gene encoding the 34-kDa xylanase. Gene..

[47] Martin KL, McDougall BM, Unkles SE, Seviour RJ (2006). The three β-1, 3-glucanases from *Acremonium blochii* strain C59 appear to be encoded by separate genes. Mycol. Res..

[48] Sellick CA, Campbell RN, Reece RJ (2008). Galactose metabolism in yeast-structure and regulation of the Leloir pathway enzymes and the genes encoding them. Int. Rev. Cell Mol. Biol..

[49] Wang D (2019). A genomic island in a plant beneficial rhizobacterium encodes novel antimicrobial fatty acids and a self-protection shield to enhance its competition. Environ. Microbiol..

[50] Hsueh YH, Somers EB, Lereclus D, Wong ACL (2006). Biofilm formation by *Bacillus cereus* is influenced by PlcR, a pleiotropic regulator. Appl. Environ. Microbiol..

[51] Xiong Q (2020). Quorum sensing signal autoinducer-2 promotes root colonization of *Bacillus velezensis* SQR9 by affecting biofilm formation and motility. Appl. Microbiol. Biotechnol..

[52] Gao SF, Wu HJ, Yu XF, Qian LM, Gao XW (2016). Swarming motility plays the major role in migration during tomato root colonization by *Bacillus subtilis* SWR01. Bio Control.

[53] Ghelardi E (2012). Contribution of surfactin and *SwrA* to flagellin expression, swimming, and surface motility in *Bacillus subtilis*. Appl. Environ..

[54] Mordini S (2013). The role of *SwrA*, *DegU* and *PD3* in *fla/che* expression in *Bacillus subtilis*. PLoS One..

[55] Li P (2023). Effects and molecular mechanism of flagellar gene *flgK* on the motility, adhesion/invasion, and desiccation resistance of *Cronobacter sakazakii*. Food Res. Int..

[56] Ciftci Y, Girinathan BP, Dhungel BA, Hasan MK, Govind R (2019). *Clostridioides* difficile *SinR*’regulates toxin, sporulation and motility through protein-protein interaction with *SinR*. Anaerobe..

[57] Milton ME, Cavanagh J (2022). The biofilm regulatory network from *Bacillus subtilis*: A structure-function analysis. J. Mol. Biol..

[58] Musik JE, Zalucki YM, Day CJ, Jennings MP (2021). Expression of the *Bacillus subtilis TasA* signal peptide leads to cell death in Escherichia coli due to inefficient cleavage by *LepB*. BBA-Bio..

[59] Kanehisa M, Goto S (2000). KEGG: Kyoto encyclopedia of genes and genomes. Nucleic Acids Res..

[60] Wei J (2023). Biocontrol mechanisms of *Bacillus velezensis* against *Fusarium oxysporum* from Panax ginseng. Biol. Control..

[61] Hofmann K (2000). *Sinorhizobium meliloti* strain 1021 *bioS* and *bdhA* gene transcriptions are both affected by biotin available in defined medium. FEMS Microbiol. Lett..

[62] Shao J (2015). Analysis and cloning of the synthetic pathway of the phytohormone indole-3-acetic acid in the plant-beneficial *Bacillus amyloliquefaciens* SQR9. Microb. Cell Factories..

[63] He P (2013). Genome sequence of the plant growth promoting strain *Bacillus amyloliquefaciens* subsp. plantarum B9601-Y2 and expression of mersacidin and other secondary metabolites. J Biotechnol..

[64] Allen LL, Heng NCK, Tompkins GR (2021). *Streptococcus salivarius* isolates of varying acid tolerance exhibit F1F0-ATPase conservation. Caries Res..

[65] Siddiqui AA, Jalah R, Sharma YD (2007). Expression and purification of *HtpX*-like small heat shock integral membrane protease of an unknown organism related to *Methylobacillus flagellatus*. J. Biochem. Biophys. Methods.

[66] Giard JC, Verneuil N, Auffray Y, Hartke A (2002). Characterization of genes homologous to the general stress-inducible gene *gls24* in *Enterococcus faecalis* and *Lactococcus lactis*. FEMS Microbiol. Lett..

[67] Kormanec J, Ševčíková B, Halgašová N, Knirschová R, Řežuchová B (2000). Identification and transcriptional characterization of the gene encoding the stress-response σ factor σH in *Streptomyces coelicolor* A3(2). FEMS Microbiol. Lett..

[68] Akhtar AA, Turner DPJ (2022). The role of bacterial ATP-binding cassette (ABC) transporters in pathogenesis and virulence: Therapeutic and vaccine potential. Microb. Pathog..

[69] Wang SC (2020). Expansion of the major facilitator superfamily (MFS) to include novel transporters as well as transmembrane-acting enzymes. BBA-Bio..

[70] Li JY, Zhao QQ, Wuriyanghan H, Yang C (2021). Biocontrol bacteria strains Y4 and Y8 alleviate tobacco bacterial wilt disease by altering their rhizosphere soil bacteria community. Rhizosphere..

