# Supplementary material for: Whole genome sequencing provides evidence for Bacillus velezensis SH-1471 as a beneficial rhizosphere bacterium in plants
Source: Sci Rep. 2023 Nov 27;13:20929. doi: 10.1038/s41598-023-48171-9 (PMC10684890; doi:10.1038/s41598-023-48171-9)
Supplement: Supplementary file 1 — Supplementary Information. [file 41598_2023_48171_MOESM1_ESM.pdf]

Whole genome sequencing provides evidence for *Bacillus velezensis* SH-1471 as a beneficial rhizosphere bacterium in plants

Yunxin Shen[1,2#] Jiacai Tang[1] Zhufeng Shi[1] Jiangyuan Zhao[3] Minggang Li[3] Nan Wang[1,2]
Yanfang Mo[1,2] Tongyu Yang[1,2] Xudong Zhou[1] Qibin Chen[2*] Peiweng Yang[1*]

1. Institute of Agricultural Environment and Resources, Yunnan Academy of Agricultural Sciences, Kunming 650204, China

2. College of Plant Protection, Yunnan Agricultural University, Kunming 655508, China

3. Yunnan Institute of Microbiology, Yunnan University, Kunming 650106, China

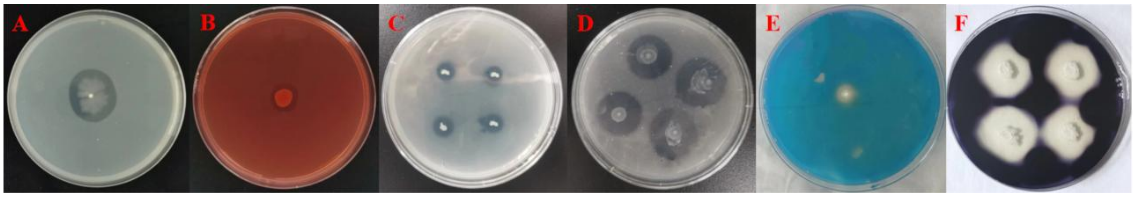

Supplementary Figure 1. Results of functional determination of strain SH-1471. A: Protease; B: Cellulase; C: Dissolved inorganic phosphorus; D: Nitrogen fixation; E: Excreting siderophore; F: secretion of amylase.

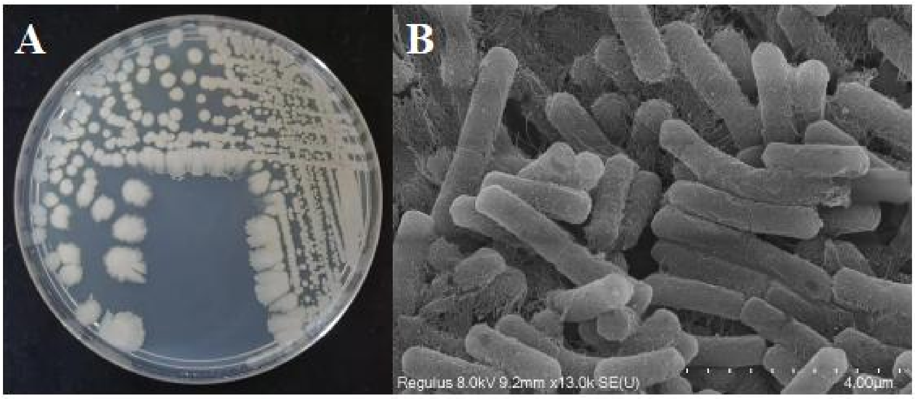

Supplementary Figure 2. Colony morphology (A) and scanning electron microscopy (B)

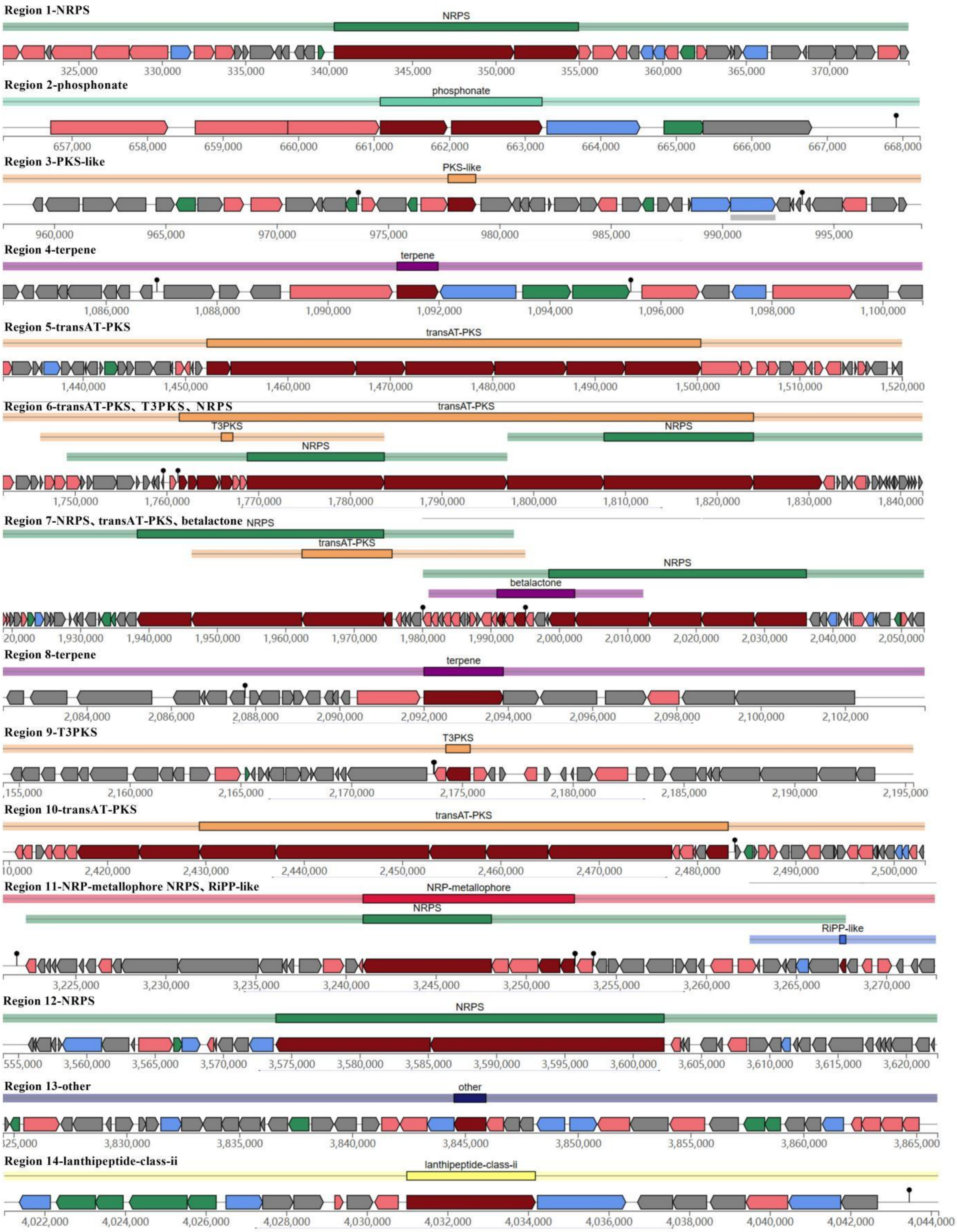

Supplementary Figure 3. Predicted results of secondary metabolite biosynthesis gene clusters in the *Bacillus velezensis* SH-1471 genome

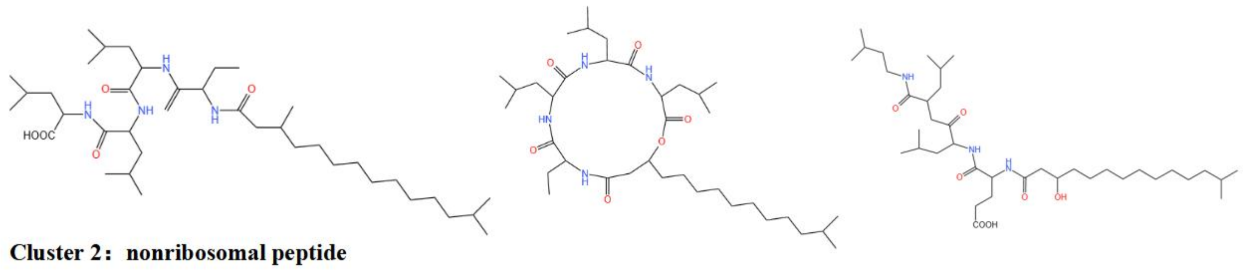

Cluster 2: nonribosomal peptide

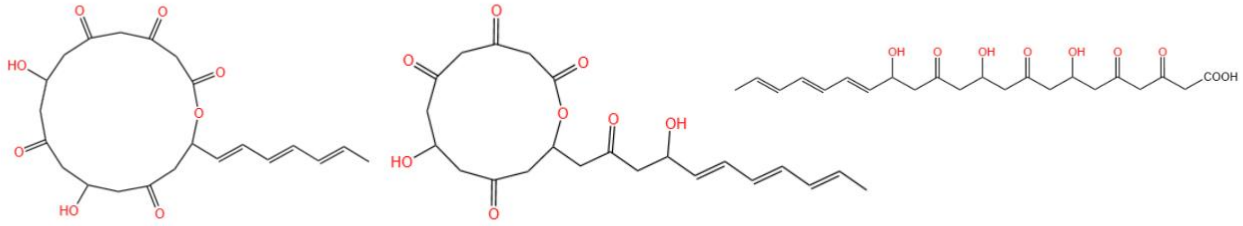

Cluster 3: polyketide

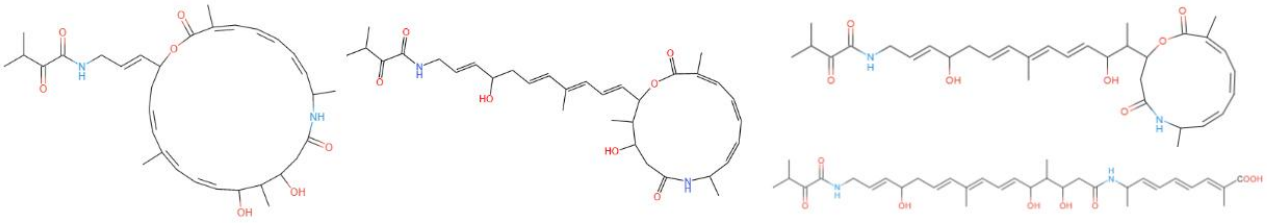

Cluster 4: polyketide, nonribosomal peptide

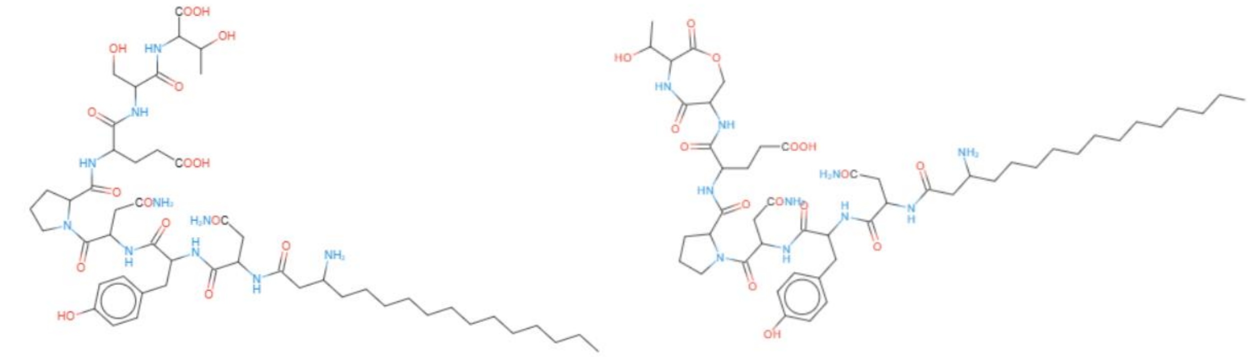

Cluster 5: polyketide, nonribosomal peptide

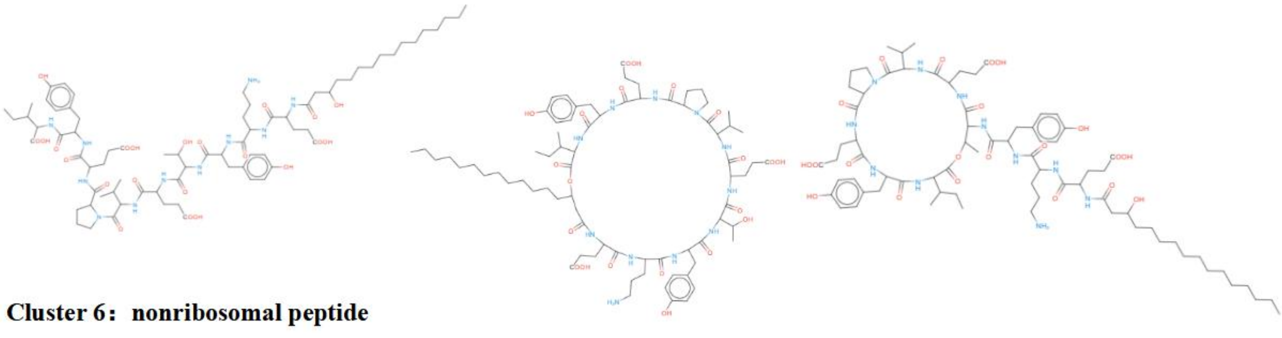

Cluster 6: nonribosomal peptide

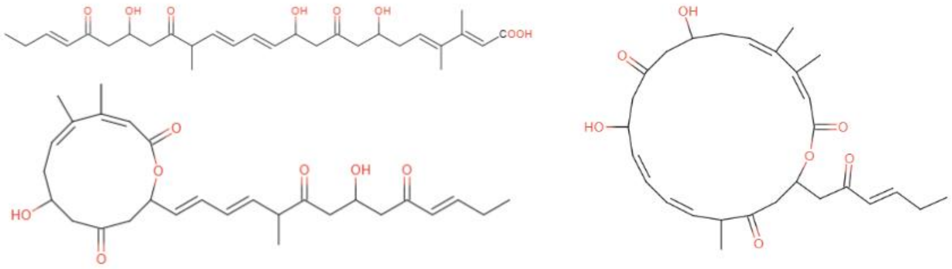

Cluster 7：polyketide

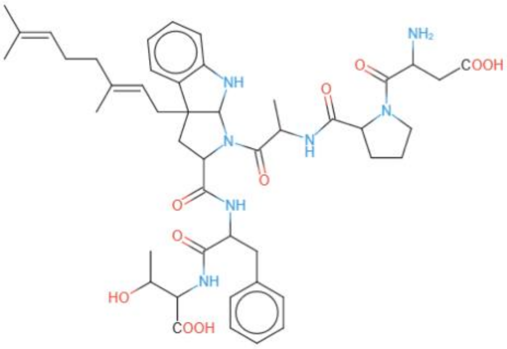

Cluster 8：ComX

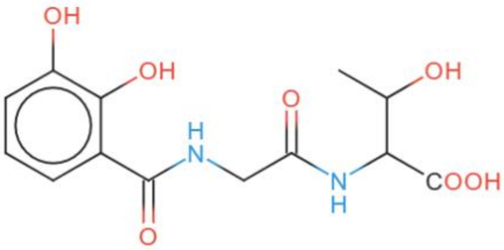

Cluster 9：nonribosomal peptide

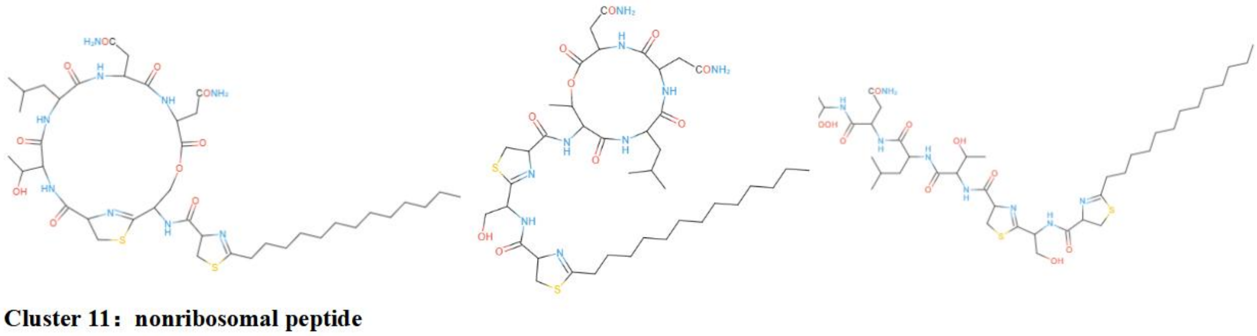

Cluster 11：nonribosomal peptide

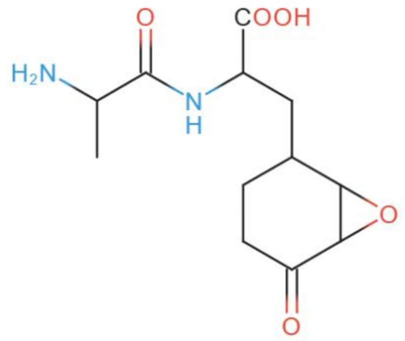

Cluster 12：Bacilysin

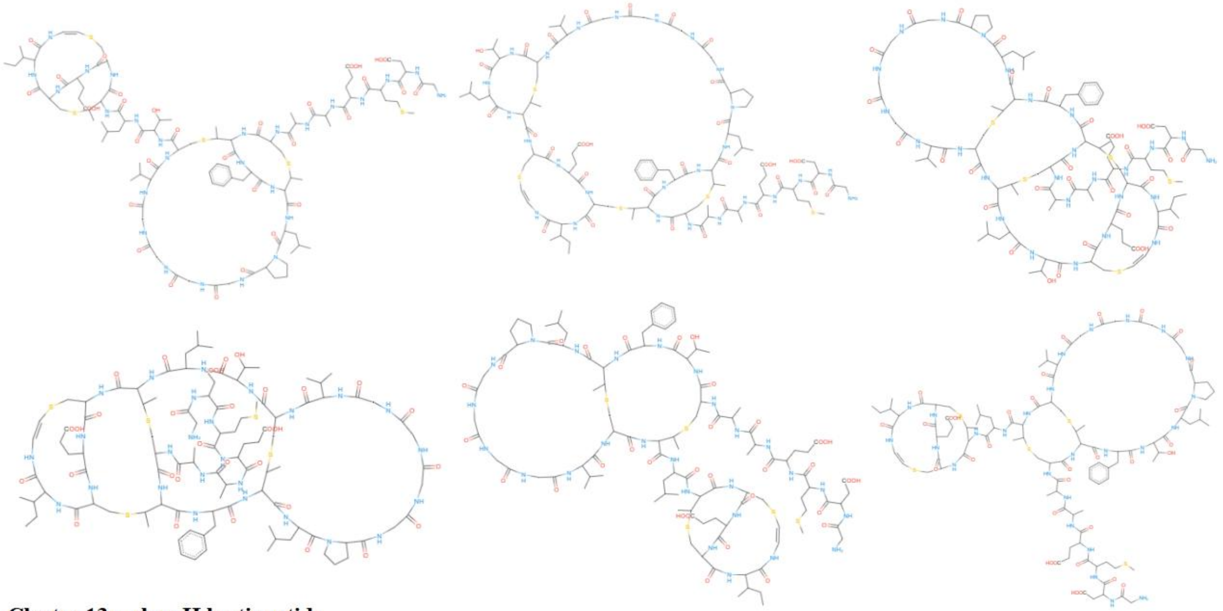

Cluster 13：class II lantipeptide

Supplementary Figure 4. Predicted structures of secondary metabolites by SH-1471 using PRISM

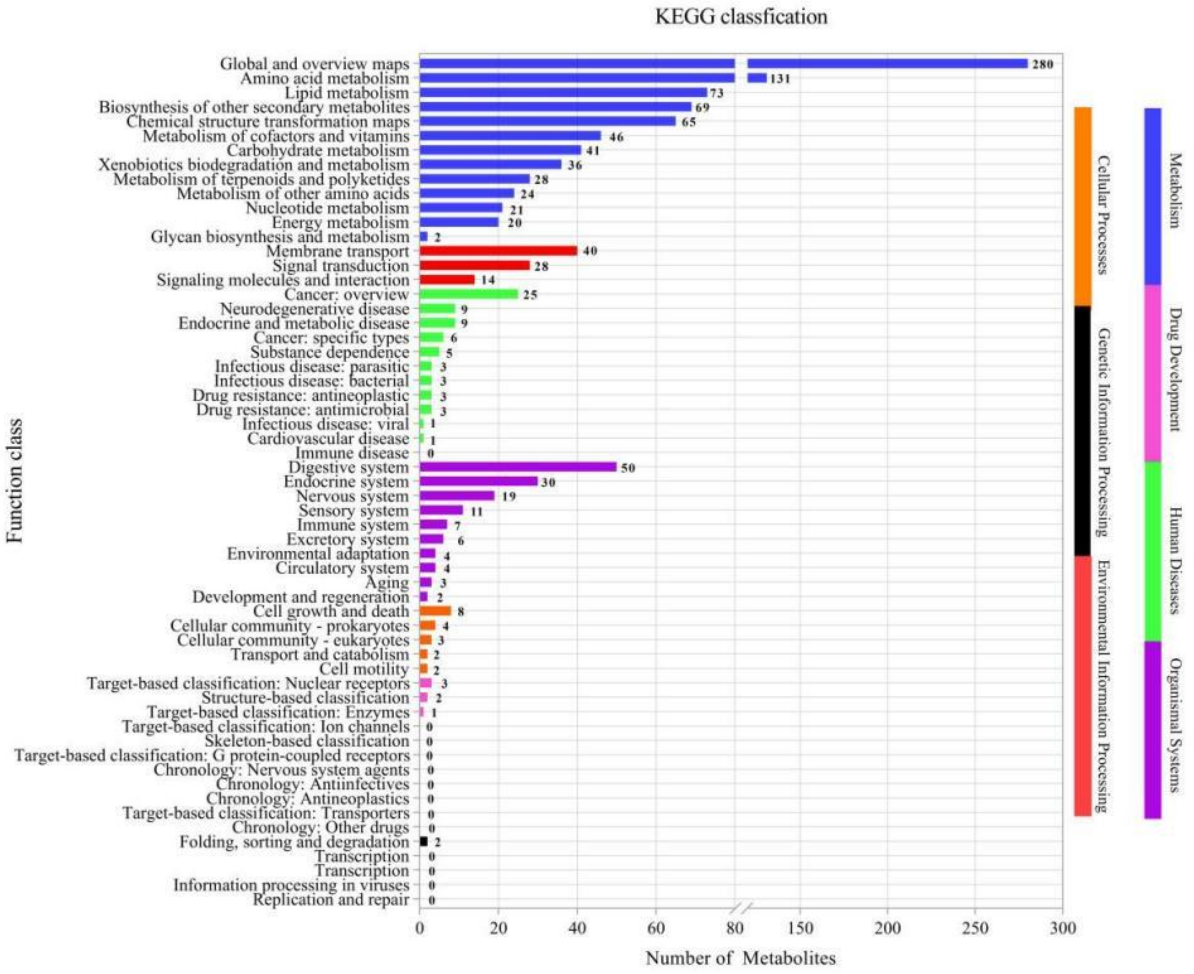

Supplementary Figure 5. Localization of metabolites in fermentation broth of *Bacillus velezensis* SH-1471 based on KEGG

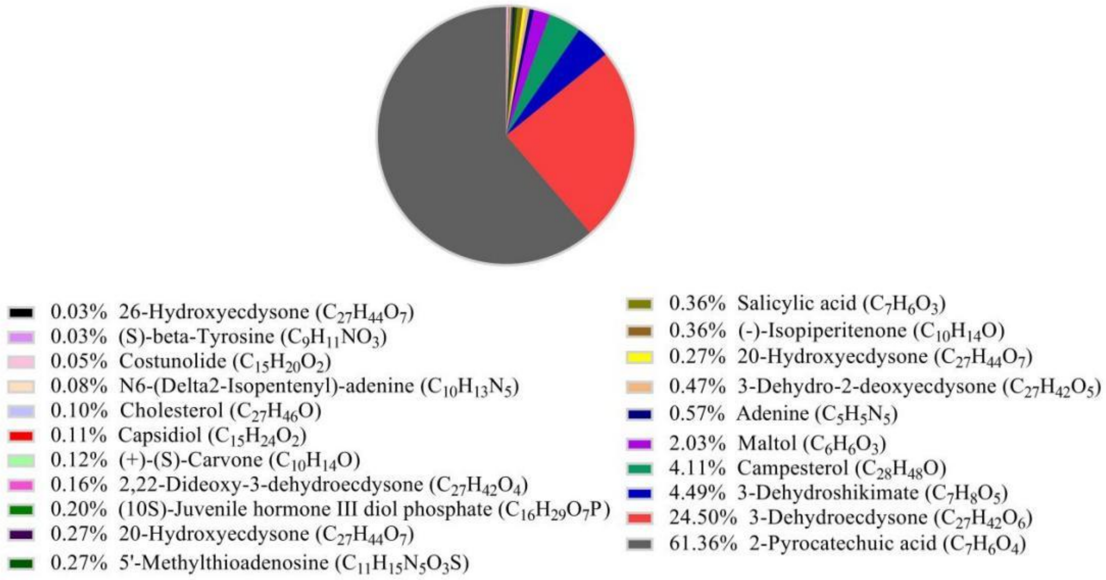

Supplementary Figure 6. Classification and proportion of metabolites in fermentation broth of *Bacillus velezensis* SH-1471 based on Metabolism of terpenoids and polyketides

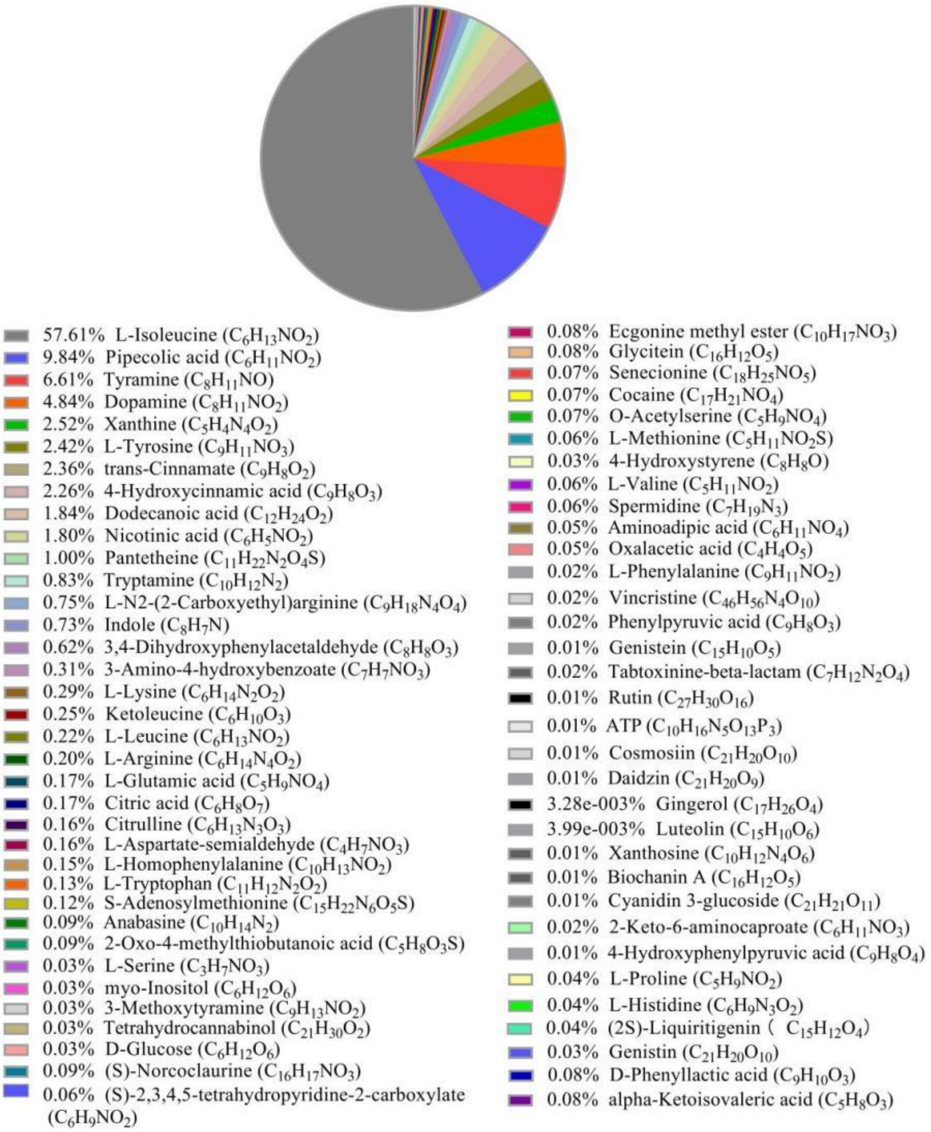

Supplementary Figure 7. Classification and proportion of metabolites in fermentation broth of *Bacillus velezensis* SH-1471 based on Biosynthesis of other secondary metabolites

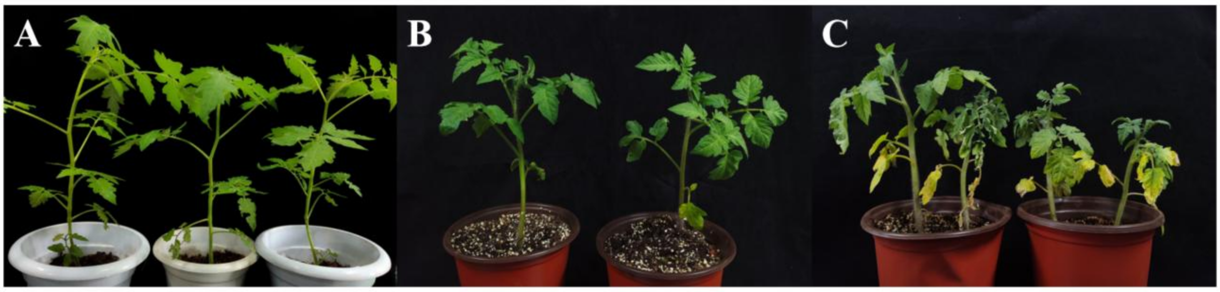

Supplementary Figure 8. Effect of functional bacteria SH-1471 on pot control of tomato Fusarium wilt (30 d). A: Control effect of SH-1471 fermentation broth on tomato fusarium wilt; B: Sterilized culture-medium; C: Pathogen+Sterilized culture-medium.

Supplementary Table 1: ANI and dDDH values of the SH-1471 genome compared to the reference genomes of the genus *Bacillus*.

Reference genome	GenBank sequence	ANI	DDH
Bacillus velezensis FZB42	CP000560.2	98.35	85.8
Bacillus velezensis SQR9	CP006890.1	98.35	84.6
Bacillus velezensis LS69	CP015911.1	98.27	85
Bacillus amyloliquefaciens B408	CP101661.1	97.70	80.1
Bacillus amyloliquefaciens DSM 7	FN597644.1	94.15	55.8
Bacillus siamensis WYJ-E14	CP101610.1	94.44	57.5
Bacillus subtilis subsp. *spizizenii* ATCC 6633	CP034943.1	77.18	20.8
Bacillus subtilis 168	CP053102.1	77.39	21.1
Bacillus subtilis TU-B-10	CP002905.1	77.35	21
Bacillus subtilis NCIB 3610	CP020102.1	77.41	21.1
Bacillus inaquosorum KCTC 13429	CP029465.1	77.53	21.3
Bacillus mojavensis UCMB5075	CP051464.1	77.64	21
Bacillus atrophaeus globigii BSS	CP007640.1	77.57	21.3
Bacillus halotolerans ZB201702	CP029364.1	77.49	20.9
Bacillus licheniformis SCDB 14	CP014842.1	73.21	19.7
Bacillus vallismortis Bac111	CP033052.1	77.53	20.8
Bacillus stercoris SMPL712	CP126678.1	77.28	20.9
Bacillus paralicheniformis Bac84	CP023665.1	72.83	20.1
Bacillus sonorensis SRCM101395	CP021920.1	72.77	19.6

Supplementary Table 2: Representative genes of *Bacillus velezensis* SH-1471 probably involved in plant bacterium interactions.

Gene	Position	Protein	Description
spo0A	2528768-2529568	Sporulation	Biofilm formation
sigH	117209-117865	RNA polymerase sigma-H factor	Biofilm formation
SigX	2356109-2356693	ECF RNA polymerase sigma factor	Biofilm formation
sinR	2562907-2563242	HTH-type transcriptional regulator	Biofilm formation
sinI	2562700-2562873	SinR antagonist	Biofilm formation
Abh	1441686-1441970	Putative transition state regulator	Biofilm formation
abrB	45545-45829	Transition state regulatory protein	Biofilm formation
sigW	200179-200742	ECF RNA polymerase sigma factor	Biofilm formation
MalK	3200010-3201611	Sensor histidine kinase	Biofilm formation
CssS	3350315-3351670	Sensor histidine kinase	Biofilm formation
resE	2356885-2358666	Sensor histidine kinase	Biofilm formation
lytS	2950687-2952468	Sensor histidine kinase	Biofilm formation
YrkQ	2664673-2665923	Sensor histidine kinase	Biofilm formation
glnK	256177-257469	Sensor histidine kinase	Biofilm formation
yfnF	743150-744061	polysaccharide biosynthesis protein	Necessary for biofilm formation
CapD	2145782-2146807	polysaccharide biosynthesis protein	Necessary for biofilm formation

tasA	2563290-2564075	Major biofilm matrix component	Necessary for biofilm formation
tapA	2564696-2565349	TasA anchoring/assembly protein	Essential for biofilm formation
sacT	3882947-3883777	Levan sucrase	Root adhesion
sacB	4136151-4137572	Levan sucrase	Root adhesion
efp	2548824-2549381	Elongation factor P	Essential for swarming motility
NusA	1704961-1706082	Transcription termination/antitermination protein	Essential for swarming motility
swrB	1690108-1690575	Swarming motility protein	Essential for swarming motility
SwrAA	3606223-3606558	Swarming motility protein	Essential for swarming motility
yhbJ	924386-925018	Multidrug efflux pump	Self-resistance against surfactin
EbrB	1838176-1838529	Multidrug efflux pump	Self-resistance against surfactin
swrC	703582-706725	Multidrug efflux pump	Self-resistance against surfactin
comP	3214904-3216190	Sensor histidine kinase	Regulator of surfactin production
srfAC	351132-354968	Surfactin synthase subunit 1	Necessary for surface motility and biofilm formation
srfAA	340328-351097	Surfactin synthase subunit 1	Necessary for surface motility and biofilm formation
srfAD	354988-355719	Surfactin synthase subunit 1	Necessary for surface motility and biofilm formation
yczE	357960-358589	Integral membrane spanning protein	Necessary for surface motility and biofilm formation
ffp	357187-357861	Phosphopantetheinyl transfease	Necessary for surface motility and biofilm formation
RemA	1614475-1614744	extracellular matrix/biofilm regulator	Protein secretion to extracellular matrix
remB	4158-4403	Extracellular matrix regulatory protein B	Protein secretion to extracellular matrix
BmrA	3535740-3537509	ABC multidrug transport	Protein secretion to extracellular matrix
ecsA	1021894-1022637	ABC multidrug transport	Protein secretion to

			extracellular matrix
ylbF	1542475-1542924	Positive regulator of ComK	Control of community development
ymcA	1750655-1751086	Hypothetical protein	Control of community development
tufA	133464-134654	Elongation factor EF-Tu	Elicitation of plant basal defence
fliD	3618934-3620454	Flagellin HAP2	Elicitation of plant basal defence
hag	3620705-3621706	Flagellin HAG	Elicitation of plant basal defence
flgK	3624118-3625635	Flagellin HAP1	Elicitation of plant basal defence
tuaA	3644023-3644682	Teichuronic acid biosynthesise	Elicitation of plant basal defence
phyC	2144314-2145465	Phytase	Phosphate availability
FecCD	187563-188570	Ferrichrome ABC transporter	Transport/binding proteins and lipoproteins
fhuG	3933736-3934749	Ferrichrome ABC transporter	Transport/binding proteins and lipoproteins
yclQ	390277-391221	Ferrichrome ABC transporter	Transport/binding proteins and lipoproteins
xynA	3746199-3746840	Endo-1,4-beta-xylanase A	Extracellular degradation of plant cell walls
xynD	1936642-1938177	Arabinoxylan arabinofuranohydrolase	Extracellular degradation of plant cell walls
YfeH	184830-185813	Hypothetical symporter	Transport of oligosaccharides
xynB	1859557-1860684	Xylan beta-1,4-xylosidase	Carbohydrate metabolism
XynC	1935319-1936590	Glucuronoxylanase	Glucan degradation
bglC	1930755-1932260	Endo-beta-1,3-1,4 glucanase	Glucan degradation
GalE	3962136-3963152	lactose metabolism repressor protein	Lactose metabolism
lacR	1249158-1249919	lactose metabolism repressor protein	Lactose metabolism
lacG	1247505-1248905	6-phospho-beta-galactosidase	Hydrolyzation of phospholactose
lacE	1245418-1247115	Phosphotransferase system	Cellobiose degradation
lacF	1247127-1247441	Phosphotransferase system	Cellobiose degradation
galK	1244233-1245402	Galactokinase galK1	Cellobiose degradation
galE	1243238-1244230	UDP-glucose 4-epimerase	Cellobiose degradation
galT	1241737-1243236	Galactose-1-phosphate uridyltransferase	Cellobiose degradation
ganA	1240573-1241685	Arabinogalactan endo-1,4-beta-galactosidase	Extracellular degradation of plant cell walls
yhfE	1038439-1039479	Glucanase/aminopeptidase	Glucan degradation

bglS	3975640-3976371	Beta-glucanase	Glucan degradation
dhaS	2090352-2091911	Putative indole-3-acet-aldehyde dehydrogenase	Trp-dependent IAA synthesis
ysnE	3890499-3890957	Putative IAA acetyl-transferase	Trp-dependent IAA synthesis
yhcX	944282-945766	Nitrilase	Trp-dependent IAA synthesis
alsD	3691124-3691891	Alpha-acetolactate decarboxylase	Synthesis of 2,3-butanediol
alsS	3691952-3693664	Acetolactate synthase	Synthesis of 2,3-butanediol
alsR	3693821-3694729	Transcriptional regulator	Regulator of the alsDS operon
pta	3839217-3840188	phosphate acetyltransferase	Strongly upregulated by root exudate

Supplementary Table 3: pH stress responsive genes in *Bacillus velezensis* SH-1471 genome.

Gene ID	Hit_description	Database
pH stress resistance		
chr_877	Na(+)/H(+) antiporter	NCBI nr
chr_551	Na(+)/H(+) antiporter NhaC	NCBI nr
chr_1159	Na(+)/H(+) antiporter YjbQ	Swiss-prot
chr_3212	Na(+)/H(+) antiporter MnhA	Swiss-prot
chr_3211	Na(+)/H(+) antiporter subunit A	Swiss-prot
chr_3212	Na(+)/H(+) antiporter subunit B	Swiss-prot
chr_3213	Na(+)/H(+) antiporter subunit C	Swiss-prot
chr_3214	Na(+)/H(+) antiporter subunit D	Swiss-prot
chr_3215	Na(+)/H(+) antiporter subunit E	Swiss-prot
chr_3216	Na(+)/H(+) antiporter subunit F	Swiss-prot
chr_3217	Na(+)/H(+) antiporter subunit G	Swiss-prot
chr_3211	cation/H(+) antiporter subunit A	NCBI nr
chr_3212	cation/H(+) antiporter subunit B	NCBI nr
chr_3213	cation/H(+) antiporter subunit C	eggNOG
chr_3214	cation/H(+) antiporter subunit D	NCBI nr
chr_3215	cation/H(+) antiporter subunit E	eggNOG
chr_3216	cation/H(+) antiporter subunit F	eggNOG
chr_3217	cation/H(+) antiporter subunit G	eggNOG
chr_3407	cation/H(+)antiporter subunit CPA1	NCBI nr
chr_990	cation/H(+)antiporter subunit CPA2	NCBI nr
chr_991	cation/H(+) antiporter	NCBI nr
chr_800	Ca(2+)/H(+) antiporter ChaA	Swiss-prot
chr_3988	Ca(2+)/H(+) symporter CitMHS	NCBI nr
chr_990	K(+)/H(+) antiporter subunit KhtU	Swiss-prot
chr_2813	K(+)/H(+) antiporter subunit KhtT	NCBI nr

chr_992	K(+)/H(+) antiporter modulator KhtS	Swiss-prot
chr_3762	F0F1 ATP synthase subunit beta	NCBI nr
chr_3767	F0F1 ATP synthase subunit C	NCBI nr
chr_3762	F0F1 ATP synthase subunit B	NCBI nr
chr_3764	F0F1 ATP synthase subunit A	NCBI nr
chr_3765	F0F1 ATP synthase subunit	eggNOG
chr_3766	ATP synthase F(0) sector subunit B	NCBI nr
chr_3769	ATP synthase protein I	NCBI nr
chr_1675	ATP synthase	eggNOG
chr_3768	F-type H+-transporting ATPase	NCBI nr
chr_3407	LI and Rb/H(+) antiporter	NCBI nr

Supplementary Table 4: Oxidative stress responsive genes in *Bacillus velezensis* SH-1471 genome.

Gene ID	Hit_description	Database
Oxidative stress resistance		
chr_3915	DsbA family oxidoreductase	NCBI nr
chr_2539	Superoxide dismutase [Fe-Mn]	NCBI nr
chr_3260	Ferredoxin--NADP reductase 2	NCBI nr
chr_1356	Glutathione reductase	NCBI nr
chr_3689	Glutathione peroxidase-like eroxiredoxin HYR1	NCBI nr
chr_2086	Glutathione reductase	Swiss-prot
chr_908	Oxidative stress	eggNOG
chr_78	Oxidative stress	eggNOG
chr_1785	Peroxiredoxin Ohr	NCBI nr
chr_2617	Oxidative stress	NCBI nr
chr_1354	Peroxiredoxin Ohr	NCBI nr
chr_4069	Peroxiredoxin Bcp	NCBI nr
chr_871	Superoxide dismutase-like protein YojM	Swiss-prot
chr_2013	Superoxide dismutase [Mn]	Swiss-prot
chr_1876	Superoxide dismutase SodA	NCBI nr
chr_2005	Superoxide dismutase [Fe]	Swiss-prot
chr_2329	Thiol-disulfide oxidoreductase ResA	Swiss-prot
chr_1876	Thioredoxin-like protein YneN	Swiss-prot
chr_3412	Thioredoxin-like protein YtpP	Swiss-prot
chr_3334	Thioredoxin-like protein YusE	Swiss-prot
chr_3545	Thioredoxin reductase OS	Swiss-prot
chr_1426	Thioredoxin	eggNOG
chr_309	Thioredoxin reductase (NADPH)	NCBI nr
chr_3053	Thioredoxin	NCBI nr
chr_444	Thioredoxin	NCBI nr
chr_2310	Thioredoxin reductase	NCBI nr
chr_4070	Thioredoxin-disulfide reductase	NCBI nr
chr_2906	Thioredoxin 1	NCBI nr
chr_4160	Thioredoxin	NCBI nr

Supplementary Table 5: Ionic and heavy metal stress responsive genes in *Bacillus velezensis* SH-1471 genome.

Gene ID<br>Ionic and heavy metal stress resistance	Hit_description	Database
chr_1279	Magnesium transporter YhiD	Swiss-prot
chr_1374	Magnesium transporter MgtE	Swiss-prot
chr_2514	Magnesium transporter CorA	NCBI nr
chr_813	Magnesium and cobalt transporter CorA	NCBI nr
chr_1368	Cobalt ABC transporter	NCBI nr
chr_156	Cobalt ABC transporter	NCBI nr
chr_154	Cobalt transporter ATP-binding subunit	NCBI nr
chr_1367	Cobalt/nickel transport system permease protein	NCBI nr
chr_643	Cobalt efflux system protein (zinc/cadmium/cobalt)	NCBI nr
chr_3032	Cobalt efflux system protein (zinc/cadmium/cobalt)	NCBI nr
chr_535	Cadmium, cobalt and zinc/H(+)-K(+) antiporter	Swiss-prot
chr_3416	Cadmium, zinc and cobalt-transporting ATPase	Swiss-prot
chr_1708	Membrane-associated zinc metalloprotease	eggNOG
chr_3178	Zinc metallopeptidase	NCBI nr
chr_1427	Zinc-transporting ATPase	Swiss-prot
chr_3831	Zinc metalloprotease YwhC	Swiss-prot
chr_1168	Zinc metalloprotease zmpB	NCBI nr
chr_3413	Zinc-dependent metalloprotease	NCBI nr
chr_76	ATP-dependent zinc metalloprotease FtsH	NCBI nr
chr_274	Zinc transport system permease protein	NCBI nr
chr_273	Zinc transport system ATP-binding protein	NCBI nr
chr_272	Zinc transport system zinc-binding lipoprotein adcA	NCBI nr
chr_3138	Manganese transport protein MntH	NCBI nr
chr_3137	Manganese/iron transport system ATP-binding protein	NCBI nr
chr_813	Metal ion transporter YfjQ	Swiss-prot
chr_422	Divalent metal cation transporter MntH	Swiss-prot
chr_388	Mn2+/Fe2-transporter NRAMP family	NCBI nr
chr_965	Fluoride ion transporter CrcB	Swiss-prot
chr_964	Fluoride ion transporter CrcB 1	Swiss-prot

Supplementary Table 6: Heat stress responsive genes in *Bacillus velezensis* SH-1471 genome.

Gene ID	Hit_description	Database
Heat stress resistance		
chr_66	heat shock protein	NCBI nr
chr_78	Heat shock protein 33 homolog	NCBI nr
chr_1389	heat shock protein HtpX	NCBI nr
chr_2583	Heat shock protein 70	NCBI nr
chr_1389	heat shock protein HtpX	NCBI nr
chr_2585	Heat shock protein DnaK	eggNOG
chr_753	Heat induced stress protein YflT	eggNOG
chr_779	Heat shock protein (HSP20)	eggNOG
chr_2584	heat shock proteins DnaK and GrpE	eggNOG
chr_2585	heat shock protein	eggNOG

Supplementary Table 7: Other stress responsive genes in *Bacillus velezensis* SH-1471 genome.

Gene ID	Hit_description	Database
Other stress resistance		
chr_59	General stress protein CTC	NCBI nr
chr_264	General stress protein 69	NCBI nr
chr_277	General stress protein 16U	Swiss-prot
chr_429	general stress protein, partial	NCBI nr
chr_1356	General stress protein 17o	NCBI nr
chr_4005	universal stress protein	NCBI nr
chr_1634	Asp23/Gls24 family envelope stress response protein	NCBI nr
chr_2474	Asp23/Gls24 family envelope stress response protein	NCBI nr
chr_3907	GlsB/YeaQ/YmgE family stress response membrane protein	NCBI nr
chr_399	General stress protein 39	NCBI nr
chr_402	General stress protein 26	NCBI nr
chr_753	General stress protein 17M	NCBI nr
chr_789	General stress protein 18	NCBI nr
chr_1993	General stress protein 16O	NCBI nr
chr_3125	General stress protein 20U	NCBI nr
chr_3190	General stress protein 13	NCBI nr
chr_3671	General stress protein 14	NCBI nr
chr_3931	General stress protein A	NCBI nr
chr_276	Stress response protein SCP2	NCBI nr
chr_974	Stress response protein NhaX	NCBI nr
chr_987	Stress response protein YhaX	NCBI nr
chr_1377	Stress response protein YkoL	NCBI nr
chr_1851	Stress response protein YvgO	NCBI nr
chr_3406	Stress response protein YvgO	NCBI nr
chr_3746	Stress response protein CsbD	NCBI nr

chr_2837	Stress response	eggNOG
chr_3356	metalloregulation DNA-binding stress protein	NCBI nr
chr_4	DNA replication/repair protein RecF	NCBI nr
chr_25	DNA replication and repair protein RecR	NCBI nr
chr_413	DNA mismatch repair protein MutT	NCBI nr
chr_986	DNA alkylation repair protein	NCBI nr
chr_2850	DNA repair protein RadC	NCBI nr
chr_98	DNA repair protein RadA	Swiss-prot
chr_2464	DNA repair protein RecN	Swiss-prot
chr_2563	DNA repair protein RecO	Swiss-prot
chr_1757	DNA mismatch repair protein MutS	Swiss-prot
chr_1758	DNA mismatch repair protein MutL	Swiss-prot
chr_4168	DNA repair enzyme AdaA	Swiss-prot
chr_682	DNA replication and repair of damaged DNA	eggNOG
chr_1860	DNA damage (SOS response), including recA and lexA	eggNOG
chr_2963	repair of DNA damaged	eggNOG
chr_4128	Serine/threonine protein kinase	NCBI nr
chr_462	Serine-protein kinase RsbW	NCBI nr
chr_719	Serine/threonine protein kinase	NCBI nr
chr_1628	serine/threonine protein kinase, bacterial	NCBI nr
chr_73	serine/threonine protein kinase	eggNOG

Supplementary Table 8: Drug resistance related genes in *Bacillus velezensis* SH-1471 whole-genome

Gene ID	Hit_description	Database
chr_259	Lincomycin efflux MFS transporter Lmr(B)	NCBI nr
chr_427	Multidrug resistance protein	eggNOG
chr_202	Multidrug ABC transporter ATP-binding protein	eggNOG
chr_216	Multidrug ABC transporter	eggNOG
chr_581	Multidrug-efflux transporter 2 regulator	eggNOG
chr_599	Multidrug resistance protein, SMR family	eggNOG
chr_613	Multidrug ABC transporter ATP-binding protein	eggNOG
chr_744	Multidrug ABC transporter ATP-binding protein	eggNOG
chr_746	MFS transporter	eggNOG
chr_830	Multidrug ABC transporter ATP-binding protein	eggNOG
chr_868	ABC-type multidrug transport system, ATPase component	eggNOG
chr_3301	Multidrug resistance protein 2	Swiss-prot
chr_976	Multidrug resistance ABC transporter ATP-binding/permease protein YheI	Swiss-prot
chr_977	Multidrug resistance ABC transporter ATP-binding/permease protein YheH	Swiss-prot
chr_1786	Multidrug resistance protein EbrB	Swiss-prot
chr_1787	Multidrug resistance protein EbrA	Swiss-prot
chr_1946	Multidrug resistance protein YoeA	Swiss-prot

chr_366	MFS-type transporter YcnB	Swiss-prot
chr_529	MFS-type transporter YdeG	Swiss-prot
chr_555	MFS-type transporter YdeR	Swiss-prot
chr_576	MFS-type transporter YdgK	Swiss-prot
chr_1012	ABC-type multidrug transport system, ATPase component	eggNOG
chr_1084	Multidrug resistance protein, MATE family	eggNOG
chr_1291	Multidrug resistance efflux transporter family protein	eggNOG
chr_1349	Multidrug resistance protein, SMR family	eggNOG
chr_1349	Multidrug resistance protein YkkC	eggNOG
chr_1425	Multidrug transporter MatE	eggNOG
chr_2019	Multidrug resistance protein NorM	eggNOG
chr_2434	Multidrug resistance protein	eggNOG
chr_3347	MFS transporter, DHA2 family, multidrug resistance protein B	eggNOG
chr_3551	Multidrug resistance ABC transporter ATP-binding/permease protein BmrA	eggNOG
chr_652	MFS-type transporter YqjV	Swiss-prot
chr_738	MFS-type transporter YfnC	Swiss-prot
chr_793	MFS-type transporter YfkL	Swiss-prot
chr_799	MFS-type transporter YfkF	Swiss-prot
chr_840	MFS-type transporter YfiU	Swiss-prot
chr_910	MFS-type transporter YhcA	Swiss-prot
chr_1110	MFS-type transporter YitZ	Swiss-prot
chr_1335	MFS-type transporter YubD	Swiss-prot
chr_1447	MFS-type transporter YkuC	Swiss-prot
chr_1951	MFS-type transporter YbcL	Swiss-prot
chr_291	MFS-type transporter yusP	eggNOG
chr_598	Multidrug resistance protein, SMR family	eggNOG
chr_909	Multidrug resistance protein MdtN	eggNOG
chr_1264	Multidrug resistance protein B like protein	eggNOG
chr_1350	Multidrug resistance protein, SMR family	eggNOG
chr_3725	MFS-type transporter YwoD	eggNOG
chr_175	MFS transporter	eggNOG
chr_282	MFS transporter	eggNOG
chr_301	MFS transporter, ACS family, D-galactonate transporter	eggNOG
chr_315	MFS transporter	eggNOG
chr_2611	MFS-type transporter YfiS	Swiss-prot
chr_2640	MFS-type transporter YybF	Swiss-prot
chr_2959	MFS-type transporter YtbD	Swiss-prot
chr_3089	MFS-type transporter YttB	Swiss-prot
chr_3347	MFS-type transporter YusP	Swiss-prot
chr_3374	MFS-type transporter YvqJ	Swiss-prot
chr_3580	MFS-type transporter YvkA	Swiss-prot
chr_3723	MFS-type transporter YwoG	Swiss-prot
chr_3859	MFS-type transporter YwfA	Swiss-prot

chr_3860	MFS-type transporter YdgK	Swiss-prot
chr_3991	MFS-type transporter YxiO	Swiss-prot
chr_4101	MFS-type transporter YxaM	Swiss-prot
chr_4142	MFS-type transporter YfmI	Swiss-prot
chr_291	EmrB/QacA subfamily drug resistance transporter	NCBI nr
chr_576	Drug resistance transporter, Bcr/CflA family	NCBI nr
chr_576	MFS transporter, DHA1 family, bicyclomycin/chloramphenicol resistance protein	NCBI nr
chr_909	Multidrug resistance protein A	NCBI nr
chr_910	MFS-type transporter ycnB	NCBI nr
chr_910	EmrB/QacA family drug resistance transporter	NCBI nr
chr_3347	EmrB/QacA family drug resistance transporter	NCBI nr
chr_3860	Drug resistance transporter, Bcr/CflA family	NCBI nr
chr_498	MFS transporter	eggNOG
chr_504	MFS transporter	eggNOG
chr_564	MFS transporter	eggNOG
chr_594	MFS transporter, DHA1 family, chloramphenicol resistance protein	eggNOG
chr_597	MFS transporter	eggNOG
chr_605	MFS transporter, SP family, major inositol transporter	eggNOG
chr_847	Multidrug MFS transporter	eggNOG
chr_850	MFS-type transporter YfhI	eggNOG
chr_1010	MFS transporter	eggNOG
chr_1062	MFS transporter, DHA1 family, multidrug resistance protein	eggNOG
chr_1297	MFS transporter	eggNOG
chr_1361	MFS transporter	eggNOG
chr_1735	MFS transporter	eggNOG
chr_1815	MFS transporter	eggNOG
chr_1897	MFS transporter	eggNOG
chr_1967	RbtT/DalT/CsbX family MFS transporter	eggNOG
chr_2538	MFS transporter	eggNOG
chr_2627	Tet(L)/Tet(K)/Tet(45) family tetracycline efflux MFS transporter	eggNOG
chr_3069	MFS transporter, AGZA family, xanthine/uracil permease	eggNOG
chr_3199	Multidrug resistance protein mdtG	eggNOG
chr_3374	MFS transporter, DHA3 family, macrolide efflux protein	eggNOG
chr_3470	MFS transporter, SP family, arabinose:H+ symporter	eggNOG
chr_3521	MFS transporter	eggNOG
chr_3650	Metabolite transport protein csbC	eggNOG
chr_3814	MFS transporter, NNP family, nitrate transporter	eggNOG
chr_3854	MFS transporter	eggNOG
chr_4046	MFS transporter, SP family, inositol transporter	eggNOG
chr_4054	Sugar porter family MFS transporter	eggNOG
chr_4066	MFS-type transporter	eggNOG
chr_4162	MFS transporter	eggNOG

Supplementary Table 9: Effect of fermentation diluent of SH-1471 on tomato growth and control effect of tomato Fusarium wilt

Treatment	Height (cm)	Stem thick (cm)	Root length (cm)	Root weight (g)	Fresh weight of above-ground part(g)	Dry weight of above-ground part(g)	Disease index	Control effect(%)
SH-1471	62.90±1.82a	2.50±0.10a	19.70±1.15a	0.84±0.19a	15.10±1.15a	3.15±0.52a	2.2±0.05a	93.8±0.18b
CK1	38.77±1.99b	1.77±0.16b	12.85±0.53b	0.53±0.04b	8.49±2.11b	2.14±0.28b	10.6±1.65c	-
CK2	28.35±2.21b	1.39±0.11c	8.99±0.26b	0.25±0.05c	6.21±0.29c	1.31±0.26c	38.3±1.58d	-

CK1: Sterilized culture-medium, CK2: Pathogen+Sterilized culture-medium. The data in the table are mean±standard deviation. Different letters indicate significant difference at 0.05 level by Duncan's new complex range test before and after fermentation condition optimization.
